# Diphosphine Bridge Control of Intramolecular Pt···Pt Association in Dinuclear Pt(II) Complexes with a Dimetalated N^∧^C^∧^C Ligand

**DOI:** 10.1021/acs.inorgchem.6c01662

**Published:** 2026-06-11

**Authors:** Salvador R. Marín-González, Dionisio Poveda, Delia Bautista, Juan Gil-Rubio, Pablo González-Herrero, Ángela Vivancos

**Affiliations:** † Departamento de Química Inorgánica, Facultad de Química, 16751 Universidad de Murcia, Campus de Espinardo, 19, Murcia 30100, Spain; ‡ Área Científica y Técnica de Investigación, Universidad de Murcia, Campus de Espinardo, 21, Murcia 30100, Spain

## Abstract

Dinuclear platinum­(II) complexes capable of establishing reversible metal–metal interactions constitute attractive platforms for stimuli-responsive luminescent systems. Here, we report a family of diphosphine-bridged complexes of general formula [{Pt­(dmtppy)}_2_(μ-P^∧^P)] incorporating a dimetalated N^∧^C^∧^C ligand derived from 2-(4,4″-dimethyl-[1,1′:3′,1″-terphenyl]-5′-yl)­pyridine, which enables systematic investigation of intramolecular Pt···Pt association. Structural studies reveal that the dppm-bridged derivative (dppm = bis­(diphenylphosphino)­methane) adopts a stacked conformation featuring a Pt···Pt interaction in solution and in the solid state. In contrast, complexes containing longer diphosphine bridges do not exhibit metallophilic contacts. NMR and electronic spectroscopic studies demonstrate that the dppm complex establishes in solution a solvent- and temperature-dependent equilibrium between open and closed conformations. These conformers give rise to distinct emissive excited states, namely, a structured ^3^ILCT/MLCT emission associated with the open form and a lower-energy ^3^MMLCT emission arising from the Pt···Pt-associated species. The interconversion between both structures leads to dual emissions whose relative contributions can be tuned by solvent and temperature. In contrast, complexes containing longer diphosphine bridges display emissions localized on the individual {Pt­(dmtppy)} fragments, with solution quantum yields influenced by linker length and flexibility. These results provide clear spectroscopic evidence for reversible intramolecular metallophilic association and highlight diphosphine-bridged architectures as promising platforms for responsive Pt­(II) emitters.

## Graphical abstract

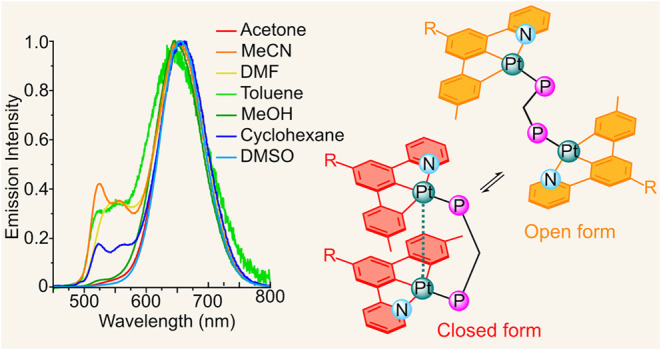

## Introduction

Luminescent platinum­(II) complexes featuring chelating heteroaromatic ligands have attracted considerable attention owing to their tunable and medium-sensitive excited states and their potential applications in optoelectronics, bioimaging, and chemical sensing.^1–3^ A key characteristic of these systems is the capacity to modulate their luminescence through the formation of molecular aggregates or supramolecular assemblies.^4–8^ These phenomena usually arise from noncovalent interactions, most commonly metallophilic Pt···Pt contacts and/or π-stacking between aromatic ligands. Such interactions significantly perturb the frontier orbitals of the complexes, enabling the formation of excimers or aggregates that typically produce red-shifted emission relative to the corresponding mononuclear species.^9,10^ Furthermore, this luminescence modulation can be affected by a number of external factors, such as the presence of organic vapors or the application of a mechanical stimulus, forming the basis for the development of functional stimuli-responsive supramolecular architectures and chemical sensors.^11–15^

With the aim of achieving a precise modulation of the luminescent properties of dinuclear Pt­(II) complexes, considerable effort has been devoted to identifying bridging ligands that allow a high degree of control over the intramolecular Pt···Pt distances. In particular, rigid linkers have been employed to enforce intramolecular metallophilic interactions between two Pt­(II) planar subunits that persist in solution, affording dinuclear complexes with well-defined structures and electronic features.^16–20^ This strategy has led to a broad family of bimetallic Pt­(II) systems incorporating terdentate ligands such as N^∧^N^∧^N (e.g., terpyridine-type ligands),^21–26^ as well as cyclometalated N^∧^N^∧^C,^27–33^ N^∧^C^∧^N,^34–38^ or C^∧^N^∧^C scaffolds.^39,40^ A variety of bridging units—including diphosphines,^27,30,32,33,39–45^ pyrazolates,^46–48^ triazolates,^49^ thiolates,^50^ bis­(NHC) ligands,^51,52^ carbolines,^53^ thiopyridyl groups,^54^ and acetylides^22,55,56^ —have been explored to tune structural rigidity and metal–metal separation.

In contrast, dinuclear Pt­(II) complexes containing flexible linkers may establish dynamic conformational equilibria in solution, enabling reversible intramolecular association between the terminal coordination fragments through Pt···Pt contacts and π interactions leading to pronounced spectral changes.^17^ Such behavior positions these species as promising metallofoldameric platforms for the development of multistimuli-responsive functional materials. Indeed, several examples incorporating terpyridyl,^27,57,58^ N^∧^N^∧^C,^57,58^ N^∧^C^∧^N,^58^ C^∧^N^∧^C,^40^ or bipyridine-type^59,60^ ligands have been reported to undergo reversible intramolecular association, with aggregation equilibria strongly dependent on bridge length, temperature, acid–base reactivity, or the composition of solvent mixtures. These controllable association–dissociation processes evoke parallels with biomolecular hairpin structures featuring reversible “sticky-end” interactions.^61,62^

However, direct spectroscopic evidence for equilibria between structurally distinct emissive conformations in diphosphine-bridged Pt­(II) systems remains poorly defined.^63^ Our recently developed photochemical strategy for the preparation of Pt­(II) complexes bearing dimetalated N^∧^C^∧^C ligands offers new opportunities to investigate such behavior,^64^ as these fragments combine strong ligand-field effects with a pronounced tendency to engage in intermolecular and intramolecular aggregation.^65^ Our initial studies demonstrate that mononuclear Pt­(II) N^∧^C^∧^C complexes can display highly efficient emission in both fluid solution and rigid media, in clear contrast to many Pt­(II) complexes bearing simple C^∧^N^∧^C ligands, which are often only weakly emissive because the mutually trans arrangement of the metalated carbon atoms tends to promote excited-state distortions that facilitate nonradiative decay.^66^

Here, we report a series of diphosphine-bridged dinuclear complexes of general formula [{Pt­(dmtppy)}_2_(μ-P^∧^P)], where dmtppy denotes dimetalated 2-(4,4″-dimethyl-[1,1′:3′,1″-terphenyl]-5′-yl)­pyridine, which have been designed to examine how the length and topology of the diphosphine bridge influence intramolecular Pt···Pt association. Structural and spectroscopic studies reveal that the dppm-bridged derivative establishes in solution a solvent- and temperature-dependent equilibrium between open and Pt···Pt-associated conformations, which give rise to distinct emissive excited states. In contrast, complexes with longer diphosphine bridges do not show intramolecular metallophilic contacts and lead to emissions localized on the individual {Pt­(dmtppy)} fragments. These findings provide direct insight into the factors governing intramolecular aggregation in dinuclear Pt­(II) systems and highlight the potential of complexes containing short and flexible diphosphine bridges as dynamically responsive luminescent platforms.

## Results and Discussion

### Synthesis and Characterization

The complex Bu_4_N­[PtCl­(dmtppy)] (**1**) was employed as precursor for the synthesis of the target dinuclear complexes [{Pt­(dmtppy)}_2_(μ-P^∧^P)] (**2**–**8**, Scheme 1), with P^∧^P = bis­(diphenylphosphino)­methane (dppm, **2**), 1,2-bis­(diphenylphosphino)­ethane (dppe, **3**), 1,3-bis­(diphenylphosphino)­propane (dppp, **4**), 1,4-bis­(diphenylphosphino)­butane (dppb, **5**), 1,6-bis­(diphenylphosphino)­hexane (dpph, **6**), bis­[(2-diphenylphosphino)­phenyl] ether (pop, **7**), and 1,1′-bis­(diphenylphosphino)­ferrocene (dppf, **8**), by reaction with the appropriate diphosphine in 2:1 molar ratio at room temperature in CH_2_Cl_2_. The complexes were obtained as red (**2**), yellow-orange (**3**, **7**, **8**), or yellow (**4–6**) solids in moderate to good yields (50–81%) after purification through recrystallization and/or column chromatography.

**Scheme 1:**
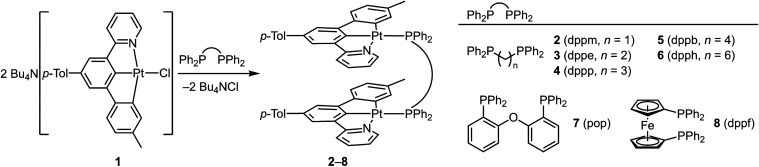
Synthesis of Complexes [{Pt­(dmtppy)}_2_(μ-P^∧^P)] (**2**–**8**)

All complexes were characterized by ^1^H, ^13^C­{^1^H}, ^195^Pt, and ^31^P­{^1^H} NMR spectroscopy, with the exception of the dppf derivative **8**, for which only ^1^H and ^31^P­{^1^H} NMR data could be obtained because of its poor solubility (Figures S1–S17). In all cases, the NMR spectra show evidence of the symmetry of the complexes, displaying a single set of resonances for the dmtppy ligands, which contrasts with previously described dinuclear complexes containing the same terdentate ligand and di-NHC bridges, which were obtained as mixtures of atropisomers due to hindered rotation about the Pt–carbene bond.^52^ The ^1^H NMR spectra show in all cases a characteristic resonance attributable to the CH ortho to the metalated carbon of the *p*-tolyl ring, which appears as a singlet between δ 6.33 and 6.54 ppm with platinum satellites (*J*_PtH_ = 67–71 Hz). The ^1^H NMR spectrum of complex **2** exhibits several distinctive features that merit detailed comment. The signal corresponding to the pyridyl H5 proton appears at an unusually low chemical shift (δ 5.71 ppm) at room temperature, in contrast to the values observed for complexes **3**–**8** (δ 6.24–6.62 ppm). Since the crystal structure of this complex (see below) reveals a stacked arrangement of the {Pt­(dmtppy)} subunits with a Pt···Pt interaction, the H5 proton is likely shielded by the aromatic π-electron density of the adjacent metalated *p*-tolyl ring (Figure S23). This observation suggests that, in solution, complex **2** predominantly adopts a closed conformation similar to that observed in the solid state, whereas complexes **3–8** adopt open or extended conformations. Further support for this interpretation was provided by NOESY experiments, which showed contacts between H atoms located on opposite dmtppy ligands in **2**, whereas similar contacts were not observed for **3** (Figures S4 and S8). The H6 proton of the pyridyl ring in **2** is also unusually shielded (δ 6.66 ppm), which can be attributed to its proximity to the π-electron density of nearby phenyl rings of the PPh_2_ groups in the sterically crowded closed conformation (Figure S23). Variable-temperature ^1^H NMR spectra of **2** were recorded in CD_3_CN and toluene-*d*_8_ at a ca. 10^–3^ M concentration to evaluate possible conformational equilibria (Figures 1 and S18). Upon increasing the temperature, the signals of both the H5 and H6 protons of the pyridyl ring shift to higher δ values (by ca. 0.5 and 0.1 ppm, respectively), indicating that the contribution of open conformations to the averaged NMR signals increases slightly with temperature. Nevertheless, the closed conformation must remain highly predominant over the examined temperature range, as these resonances remain significantly shielded relative to those observed for complexes **3**–**8**. Consistent with this behavior, although the closed conformer contains two diastereotopic PPh_2_ groups, all P-bound phenyl rings appear equivalent at room temperature. Because equivalence of the phenyl rings requires a 180° rotation of the {Pt­(dmtppy)} subunits, a fast conformational exchange on the NMR time scale must occur, involving transient separation of the {Pt­(dmtppy)} subunits (Scheme 2). This process becomes slower upon decreasing the temperature, giving rise to the expected splitting of the phenyl signals below 238 K in CD_3_CN (Figure 1). Additional splittings were observed at lower temperatures in toluene-*d*_8_, attributable to the slowing of P–Ph rotation (Figure S18). Possible aggregation effects were also evaluated by recording the ^1^H NMR spectrum of **2** at concentrations between 1 × 10^–4^ M and 1 × 10^–3^ M in CD_3_CN (Figure S19) and between 1 × 10^–4^ M and 4 × 10^–3^ M in toluene-*d*_8_ (Figure S20), but no changes were observed, indicating that the unusual chemical shifts of the H5 and H6 resonances are not caused by intermolecular association.

**Figure 1:**
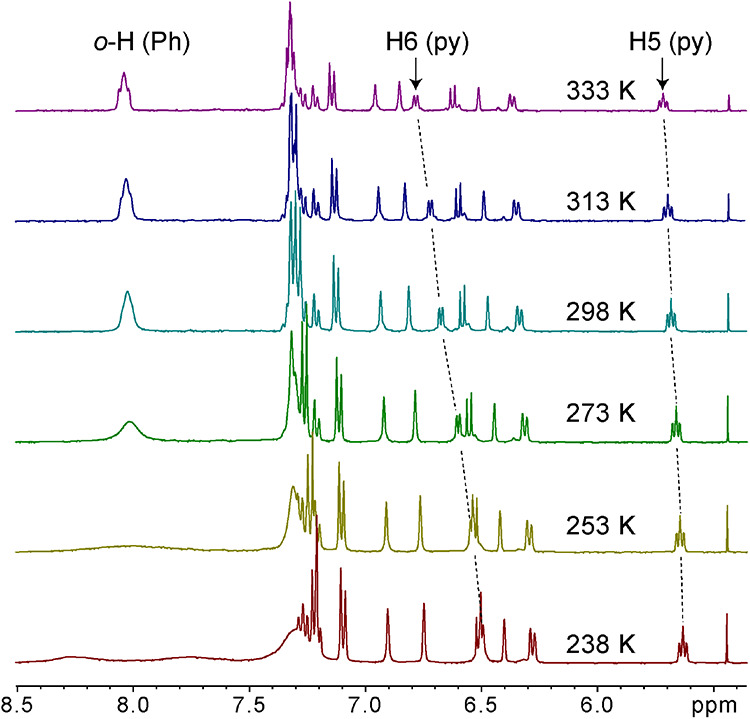
^1^H NMR spectra of complex **2** at different temperatures (CD_3_CN, 400 MHz).

**Scheme 2:**
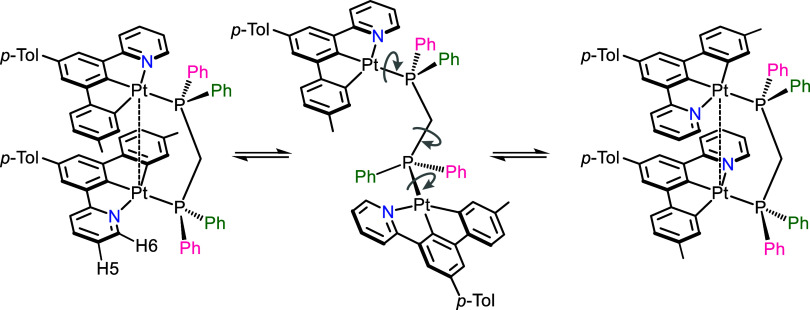
Conformational Equilibrium of Complex **2**

In all cases, the equivalence of the platinum nuclei is evidenced by the presence of a single resonance in the ^195^Pt NMR spectra, which appears as a doublet between −4183 and −4142 ppm with ^1^ *J*_PPt_ = 2040–2090 Hz. The ^13^C NMR spectra show a downfield doublet at 178.2–179.1 ppm (*J*_CP_ ∼ 100 Hz), which is characteristic of the metalated carbon atom of the central aromatic N^∧^C^∧^C ring trans to the phosphine ligand. The ^31^P­{^1^H} NMR spectra of complexes **4**–**8** show a singlet with platinum satellites between 26.8 and 34.8 ppm. The complexes bearing the phosphines with the shortest linkers, dppm (**2**) and dppe (**3**), give rise to more intricate spectral features. In these cases, the principal resonance appears as a singlet at 29.0 ppm for **2** and 31.0 ppm for **3**, accompanied by second-order patterns attributable to species incorporating either one (AA′ component of an AA′X spin system) or two (AA′ component of an AA′XX′ spin system) ^195^Pt nuclei (Figures S2 and S6). These spectra were simulated using the *g*NMR software (Figures S21 and S22), which allowed the determination of the following coupling constants: ^1^ *J*_PtP_ = 2063 Hz, ^2+3^ *J*_PtP_ = 32.0 Hz, ^3^ *J*_PP′_ = 96.6 Hz, ^4^ *J*_PtPt′_ = 140 Hz (complex **2**); ^1^ *J*_PtP_ = 2063 Hz, ^4^ *J*_PP′_ = 47.7 Hz (complex **3**). In the case of **3**, neither a four-bond Pt–P coupling nor a Pt–Pt coupling was observed, which can be rationalized by the larger separation between the involved nuclei. Additional discussion on the data extracted from the simulation of the ^31^P­{^1^H} NMR spectra of **2** and **3** is included in the Supporting Information.

The electrospray mass spectra confirm the proposed structure, showing peak clusters corresponding to the monoprotonated molecular cations ([M + H]^+^) and/or to fragment ions resulting from the loss of a {Pt­(dmtppy)} unit, ([M – {Pt­(C_25_H_19_N)} + H]^+^).

The crystal structures of complexes **2** and **3** were solved by X-ray diffraction (Figures 2 and 3, respectively). A selection of bond distances and angles is compiled in the Supporting Information (Tables S2 and S3). The structure of complex **2** shows two crystallographically inequivalent, highly planar {Pt­(dmtppy)} subunits (RMSD from plane: 0.016, 0.075 Å, excluding the pendant *p*-tolyl group), placed one above the other and nearly parallel to each other (angle between mean planes: 0.5°). They adopt opposite relative orientations (considering the C–Pt–N axes) and are slightly offset with respect to each other (torsion angle P1–Pt1···Pt2–P2: 22.4°). The coordination environments around the metal centers are also highly planar, with RMSDs of 0.026 or 0.058 Å. The Pt atoms establish a metallophilic interaction (Pt···Pt distance: 3.570 Å), which is appreciably longer than that found for stacked pairs of mononuclear complexes [Pt­(dmtppy)­(L)], with L = CO or XyNC, likely because in these cases the molecules adopt mutually antiparallel orientations, avoiding the steric repulsions between the *p-*tolyl substituents.^65^ The metal–metal distance is also longer than that found for bimetallic Pt­(II) complexes of general formula [{Pt­(N^∧^N^∧^C)}_2_(μ-dppm)], with Pt···Pt distances between 3.165 and 3.374 Å,^28,29^ with the exception of a complex with an aza-15-crown-5 substituent on the central pyridine ring, for which a long Pt···Pt separation of 3.827 Å was found.^27^ The average separation between the {Pt­(dmtppy)} mean planes in **2** is 3.400 Å, but intramolecular π interactions could not be unequivocally identified from the geometrical analysis. In contrast, intermolecular π interactions were identified between inversion-related molecules involving the pyridine ring of one of the {Pt­(dmtppy)} subunits (centroid-centroid distance: 3.431 Å, distance between the centroid of one ring and the mean plane of the other: 3.288 Å, shift distance: 0.979 Å; Figure S24).

**Figure 2:**
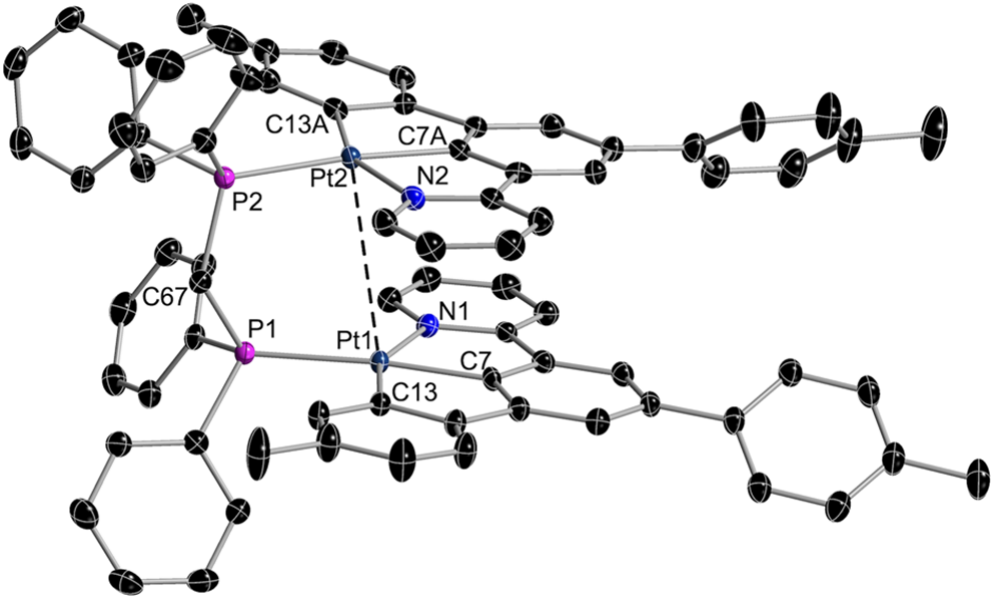
Structure of complex **2** in the crystal (thermal ellipsoids at 50% probability). Hydrogen atoms are omitted. Selected bond distances (Å) and angles (°): Pt(1)–C(7): 1.979(3); Pt(1)–C(13): 2.051(3); Pt(1)–N(1): 2.169(2); Pt(1)–P(1): 2.3354(7); Pt(2)–C­(7A): 1.974(3); Pt(2)–C­(13A): 2.046(3); Pt(2)–N(2): 2.169(3); Pt(2)–P(2): 2.3216(8); P(1)–C(67): 1.856(3); N(1)–Pt(1)–C(7): 77.45(11); C(7)–Pt(1)–C(13): 79.50(12); P(1)–C(67)–P(2): 120.11(16).

**Figure 3:**
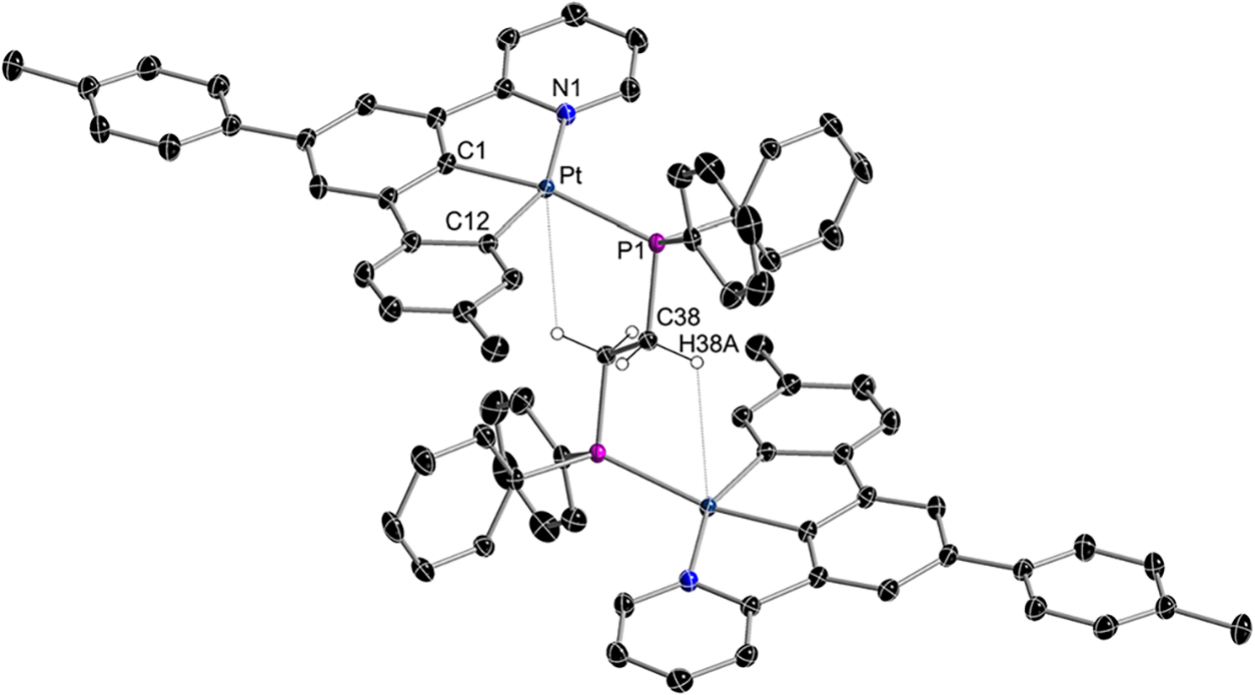
Structure of complex **3** in the crystal (thermal ellipsoids at 50% probability). Hydrogen atoms are omitted, except for those that participate on Pt···H contacts. Selected bond distances (Å) and angles (°): Pt–C(1): 1.9729(18); Pt–N(1): 2.1514(16); Pt–C(12): 2.0369(18); Pt–P(1): 2.3203(5); P(1)–C(38): 1.8408(19); C(1)–Pt–N(1): 78.01(7); C(1)–Pt–C(12): 79.98(8); C(1)–Pt–P(1): 167.25(6); C(38)–P(1)–Pt: 107.71(6).

Complex **3** crystallizes with half a molecule in the asymmetric unit; the complete molecule is generated via an inversion center situated at the midpoint of the ethylene bridge. The {Pt­(dmtppy)} subunits adopt a mutually antiparallel orientation and exhibit a more pronounced deviation from planarity (RMSD: 0.102 Å) relative to complex **2**. Similarly, the platinum coordination geometry shows increased distortion (RMSD: 0.116 Å). These geometric constraints likely arise from steric repulsion between the phenyl substituents of the dppe ligand and the dmtppy framework. The relative orientation of the {Pt­(dmtppy)} subunits appears to be caused by the especially stable all-anti conformation of the dppe ligand and the establishment of intramolecular C–H···Pt hydrogen bonds involving a methylene proton from the bridge (H38A···Pt distance: 2.894 Å). The resulting bridge conformation precludes both intramolecular Pt···Pt contacts and π-stacking interactions within the discrete molecule. In the extended structure, however, intermolecular π interactions exist between the pyridine ring and the central phenyl ring of neighboring {Pt­(dmtppy)} subunits (centroid-centroid distance: 3.767 Å, distance between the centroid of one ring and the mean plane of the other: 3.441 Å, shift distance: 1.531 Å; Figure S25), leading to infinite stacks along the *b* axis.

### Photophysical Properties

The electronic absorption spectra of dinuclear complexes **2**–**7** were recorded in MeCN at 298 K and are shown in Figure 4a; the corresponding spectroscopic data are collected in Table 1. Complex **8** was excluded from the photophysical study owing to its low solubility. All complexes display very similar spectral profiles, characterized by four main absorption bands centered at approximately 274, 322, 379, and 424 nm. The two lowest-energy bands are assigned to singlet intraligand charge-transfer (^1^ILCT) transitions with significant metal-to-ligand charge-transfer (^1^MLCT) character, in agreement with previous reports on Pt­(II) complexes containing the dmtppy ligand.^65^ In addition, complex **2** exhibits a weak, low-energy absorption band centered at 502 nm, which can be attributed to a charge-transfer transition arising from the Pt···Pt interaction to π* orbitals of the dmtppy ligand, i.e., a ^1^MMLCT [dσ* → σ­(π*)] transition. This feature indicates that the metallophilic interaction is retained in MeCN solution. The absorption spectra of **2** recorded at concentrations ranging from 2.0 × 10^–6^ to 1.0 × 10^–4^ M (Figure S26) show that the ^1^MMLCT band exhibits a measurable concentration dependence. The band remains clearly observable even at the lowest measurable concentration, consistent with an intramolecular origin of the interaction. However, its molar extinction coefficient decreases upon dilution, consistent with a shift of the conformational equilibrium toward the open form. A related behavior has previously been reported for di- and trinuclear Pt­(II) complexes^63,67^ and may be explained in terms of preferential solvation of the more extended conformations.

**Figure 4:**
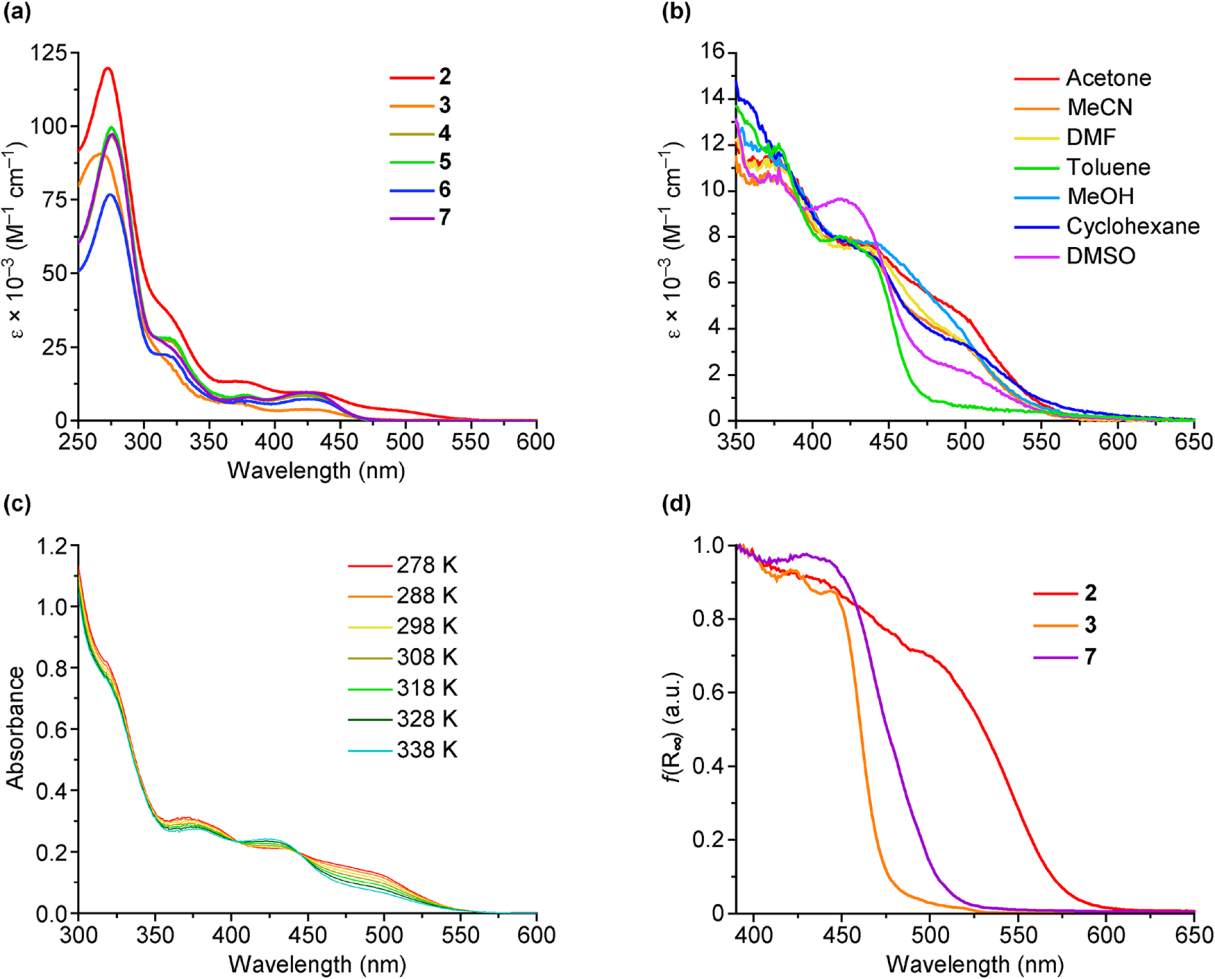
(a) Absorption spectra of complexes **2–7** in a MeCN solution at 298 K (ca. 2.5 × 10^–5^ M for **2**, **4**, **5**, and **7**; ca. 1.0 × 10^–5^ M for **3** and **6**). (b) Absorption spectra of **2** in different solvents at 298 K (ca. 1.0 × 10^–5^ M). (c) Absorption spectra of **2** in a MeCN solution (ca. 2.5 × 10^–5^ M) at different temperatures. (d) Diffuse reflectance spectra of complexes **2**, **3**, and **7** (Nujol mulls) transformed using the Kubelka–Munk function, *f*(*R*_∞_).

**Table 1:** Electronic Absorption Data of Complexes **2-7** (ca. 2.5 × 10^–5^ M for **2**, **4**, **5**, and **7**; ca. 1.0 × 10^–5^ M for **3** and **6**) in a MeCN Solution at 298 K

complex	λ_max_ /nm (ε × 10^–3^/M^–1^ cm^–1^)
**2**	272 (120), 322 (34), 376 (13), 431 (10), 501 (3)
**3**	270 (91), 323 (18), 378 (6), 424 (4)
**4**	275 (96), 322 (27), 379 (8), 423 (9)
**5**	275 (99), 322 (28), 379 (9), 423 (9)
**6**	274 (77), 322 (22), 379 (6), 425 (8)
**7**	276 (97), 321 (25), 380 (8), 423 (10)

The absorption spectrum of **2** was also recorded in several additional solvents at a concentration of 1.0 × 10^–5^ M and is compared with the spectrum in MeCN at the same concentration in Figure 4b. The corresponding data are summarized in Table S4. The molar absorptivity of the ^1^MMLCT band undergoes significant variations, suggesting the existence of a solvent-dependent equilibrium between the closed and open conformations of complex **2**, as inferred from the ^1^H NMR data (Scheme 2). This band is very weak and significantly red-shifted in toluene, probably as a consequence of π interactions between the solvent and the aromatic rings of the dmtppy ligand, which may modify both the energy and molar absorptivity of the ^1^MMLCT transition in the closed conformation. For further analysis, absorption spectra of **2** were recorded in MeCN and DMF over the temperature range 278–338 K (Figures 4c and S27). In both solvents, the absorbance of the ^1^MMLCT band (500 nm) decreased with increasing temperature, indicating a shift in the equilibrium toward the open conformation. A concomitant decrease in absorbance at 376 nm was also observed, resulting in a spectral profile that increasingly resembles those of complexes **3**–**7**, which do not exhibit intramolecular association between the {Pt­(dmtppy)} subunits.

Diffuse reflectance spectra were recorded for complexes **2**, **3**, and **7**. These compounds were selected on the basis of their red (**2**) or yellowish-orange (**3** and **7**) solid state coloration, which may indicate aggregation effects. The reflectance data were transformed using the Kubelka–Munk function and normalized to allow direct comparison (Figure 4d). In the case of complex **2**, the absorption band is markedly shifted to lower energies, consistent with the presence of Pt···Pt interactions in the solid state, giving rise to a ^1^MMLCT absorption. By contrast, complexes **3** and **7** exhibit only modest red shifts relative to their solution spectra, in agreement with the formation of molecular aggregates mediated by π interactions, as evidenced by the crystal structure of **3**.

The emission properties of complexes **2**–**7** were studied in MeCN solution, polystyrene (PS) matrices (2 wt %) and solid state at 298 K. The emission data are compiled in Table 2. Excitation and emission spectra are included in the Supporting Information (Figures S28–S30).

**Table 2:** Emission Data of Complexes **2**–**7** ^a^

**complex**	**medium**	**λ**_**em**_ ^b^ **(nm)**	**Φ** ^c^	**τ** (**μ**s)^d^
**2**	MeCN	525, 555, *655*	0.28	3.1 (529 nm)
				1.2 (655 nm)
	polystyrene	523, *645*	0.76	9.1 (655 nm)
	solid	665	0.25	1.6 (30%), 22 (70%)^e^
**3**	MeCN	*525*, 555	0.44	2.4
	polystyrene	*527*, 562	0.42	11
	solid	527, *571*, 617	<0.01	
**4**	MeCN	*525*, 555	0.08	0.6
	polystyrene	*525*, 559	0.45	12
	solid	536, *570*	0.01	
**5**	MeCN	*525*, 555	0.08	5.9
	polystyrene	525, 559	0.36	12
	solid	537, *568*	<0.01	
**6**	MeCN	*525*, 555	0.12	5.2
	polystyrene	*525*, 559	0.33	11
	solid	537, *568*	<0.01	
**7**	MeCN	*525*, 555	0.21	3.1
	polystyrene	*524*, 557	0.51	11
	solid	537, *566*	<0.01	

a The excitation wavelength was 430 nm for all measurements.

b The most intense peaks are italicized.

c Emission quantum yield.

d Lifetime: recorded at the highest-energy emission peak, unless otherwise noted.

e Biexponential decay.

The dinuclear complexes **3**–**7** exhibit highly similar, vibronically structured emission bands with a maximum at 525 nm, both in solution (Figure 5a) and in PS matrices (Figure S29). These emissions are attributed to a ^3^ILCT/MLCT excited state localized on the {Pt­(dmtppy)} subunits on the basis of previous assignments for mononuclear derivatives [Pt­(dmtppy)­(L)][Bibr ref65] and the complex [Pt­(N^∧^C^∧^C)­(PPh_3_)], with N^∧^C^∧^C = dimetalated 2-(1,1′-biphenyl-3-yl)­pyridine.^68^ The excitation spectra faithfully reproduce the absorption profiles, indicating that the emissions stem from the complexes and not from possible impurities (Figures S28 and S29). To evaluate possible aggregation effects, the emission spectrum of **5** in MeCN solution was registered at concentrations between 2.0 × 10^–6^ and 2.0 × 10^–5^ M, but no variations were observed (Figure S31). Higher concentrations could not be examined because of the limited solubility of the complex. Complexes **3**, **4**, **6**, and **7** exhibit similar or lower solubilities in MeCN, which likewise prevented measurements at higher concentrations. The measured lifetimes are consistent with triplet emissions. Notably, while substantial photoluminescence quantum yields are obtained for **3**–**7** in PS matrices (Φ = 0.33–0.51), the efficiencies in solution are markedly lower and strongly dependent on the nature of the bridging ligand. This behavior is ascribed to the capacity of the linker to either restrict or promote intramolecular encounters between the {Pt­(dmtppy)} subunits, which can enhance nonradiative decay via triplet–triplet annihilation. A comparable effect has been reported for dinuclear systems of the type [{Pt­(dmtppy)}_2_{μ-(Im_Me_)_2_(CH_2_)_*n*_}] (Im_Me_ = *N*-methylimidazol-*N*-yl-2-ylidene), featuring di-N-heterocyclic carbene bridges with aliphatic spacers of variable length,^52^ and other dinuclear complexes with flexible spacers.^59^ In this context, the relatively high emission efficiency of complex **3** in solution (Φ = 0.44) may stem from its ability to maintain spatial separation between the {Pt­(dmtppy)} units, owing to the conformational rigidity and preferred all-anti arrangement of the dppe linker, which is likely preserved to a significant extent in fluid media. A related structural effect may account for the moderate quantum yield observed for complex **7** (Φ = 0.21). In contrast, the greater conformational flexibility of the dppp, dppb, and dpph ligands in complexes **4**–**6** likely facilitates internal collisions between the {Pt­(dmtppy)} subunits, resulting in lower solution quantum yields (Φ = 0.08–0.12). In the solid state, the emissions of **3**–**7** are generally weak and moderately red-shifted relative to those observed in solution and PS matrices (Figure 5b). For complex **3**, an additional low-energy emission band appears at 617 nm, attributable to aggregation via intermolecular π interactions, as inferred from the analysis of the crystal structure.

**Figure 5:**
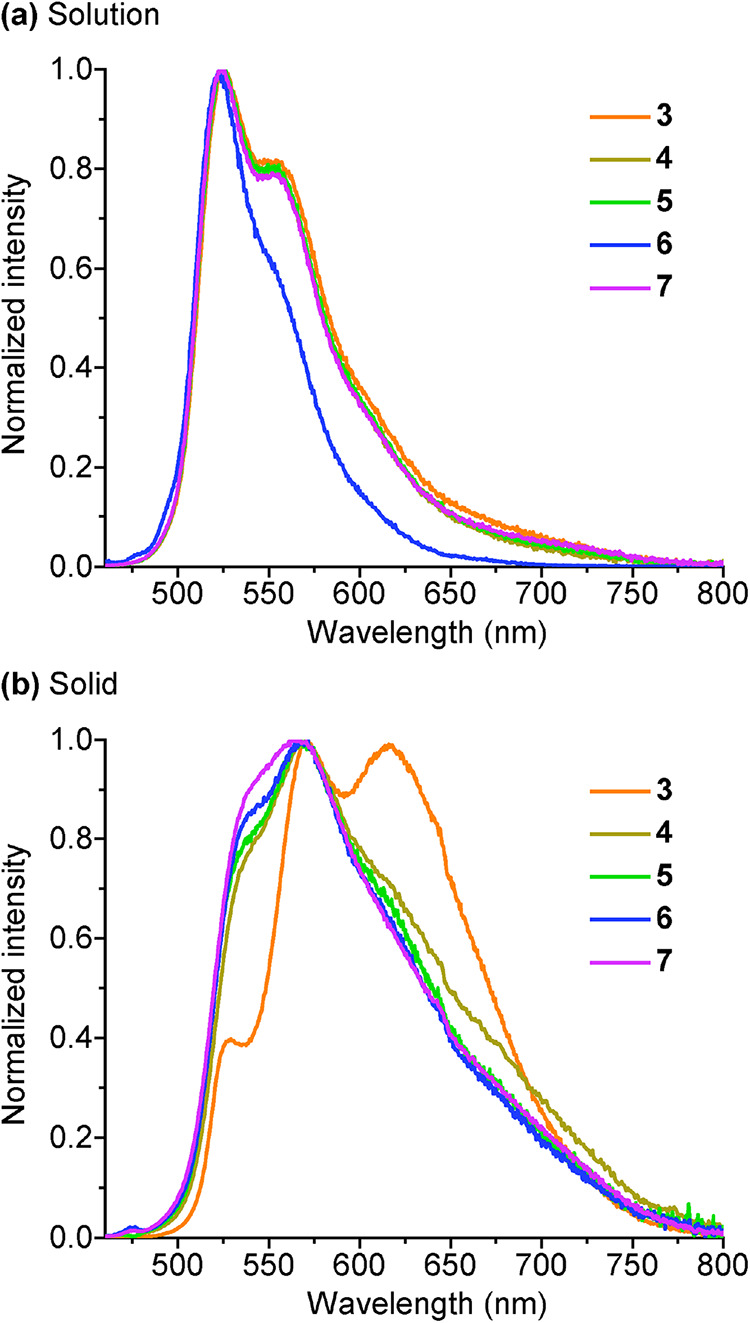
Emission spectra of complexes **3**–**7** in (a) MeCN solution (ca. 1.0 × 10^–5^ M for **3** and **6**; ca. 2.5 × 10^–5^ M for **4**, **5**, and **7**) and (b) solid state at 298 K.

Complex **2** exhibits dual emission in MeCN solution (1.0 × 10^–5^ M) and in a PS matrix when excited at 430 nm, consisting of a structured high-energy band consistent with the ^3^ILCT/MLCT emission observed for the other complexes, and a low-energy, unstructured band centered at 655 nm in solution and 645 nm in PS (Figures 6 and S29), which can be attributed to a ^3^MMLCT state. The latter predominates in both media, although its relative intensity is higher in the rigid PS matrix. In the solid state, only the ^3^MMLCT band is observed, with a maximum at 665 nm (Figure S30). The excitation spectra monitored at the ^3^MMLCT and at the ^3^ILCT/MLCT emission maxima in MeCN are clearly distinct and do not exactly reproduce the absorption profile (Figure 6). Detailed analysis indicates that the absorption spectrum combines contributions from both excitation profiles. Specifically, excitation monitored at the ^3^MMLCT emission clearly reveals the low-energy ^1^MMLCT absorption band, whereas the excitation spectrum monitored at the ^3^ILCT/MLCT emission closely resembles the absorption profiles of complexes **3**–**7**, displaying a higher relative intensity of the band at 431 nm as compared to that at 376 nm and lacking the ^1^MMLCT feature. In addition, excitation within the ^1^MMLCT absorption (500 nm) exclusively produced the ^3^MMLCT emission. These results support the coexistence of open and closed conformations of complex **2**, as previously inferred from the absorption and NMR data. Lifetimes were determined for both emission bands in MeCN solution, with the ^3^MMLCT state displaying a shorter lifetime. This behavior can be rationalized by enhanced spin–orbit coupling arising from the involvement of two Pt­(II) centers, which leads to faster radiative decay rates.^41,69^ Lifetimes in the PS matrix and the solid state are longer due to the suppression of nonradiative decay pathways associated with molecular motion. Notably, complex **2** exhibits the highest photoluminescence quantum yields of the series in both the PS matrix and the solid state, which can be attributed to the enhanced structural rigidity arising from the establishment of Pt···Pt metallophilic interactions.^17^

**Figure 6:**
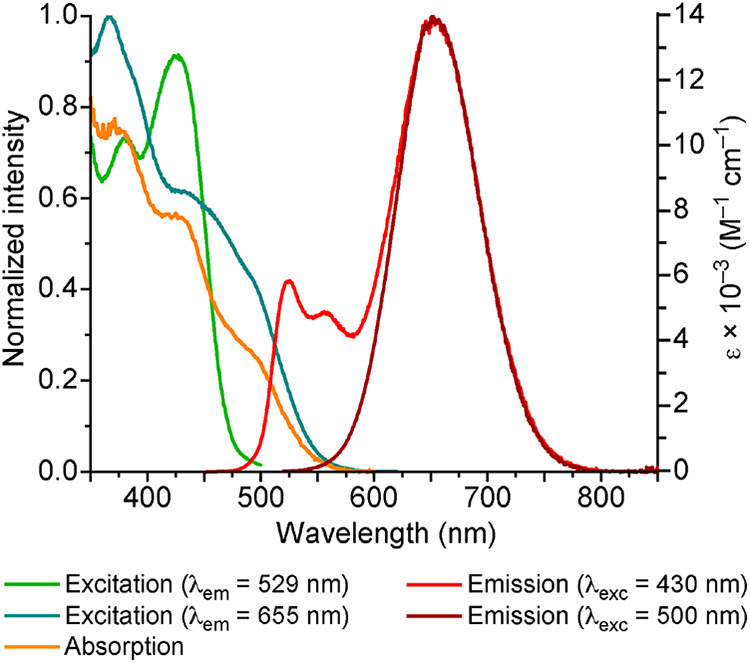
Excitation, absorption and emission spectra of complex **2** in a MeCN solution at 298 K (1.0 × 10^–5^ M).

The emission behavior of complex **2** was further investigated in MeCN solution at concentrations ranging from 2.0 × 10^–6^ to 1.0 × 10^–4^ M and in a range of other solvents at a fixed concentration of 1.0 × 10^–5^ M (Figures 7 and S32; Table 3). The ^3^ILCT/MLCT emission predominates in MeCN at 2.0 × 10^–6^ M (λ_exc_ = 430 nm), whereas the ^3^MMLCT becomes almost exclusive at 1.0 × 10^–4^ M. This behavior is consistent with the variations observed in the absorption spectra (Figure S26). It also indicates that the open conformation represents a minor contribution at a 1.0 × 10^–4^ M concentration, as deduced from the ^1^H NMR spectrum in CD_3_CN. Dual emission was observed in most of the other solvents (λ_exc_ = 430 nm), although the relative contributions of the ^3^ILCT/MLCT and ^3^MMLCT bands, as well as the overall photoluminescence quantum yields, varied significantly. As in MeCN, excitation within the ^1^MMLCT absorption band produced only the ^3^MMLCT emission in all cases (Figure S32). Likewise, the excitation spectra monitored at the ^3^MMLCT emission maximum display the ^1^MMLCT band, whereas those monitored at the ^3^ILCT/MLCT emission resemble the absorption profiles of complexes **3**–**7** (Figure S32). Cyclohexane and acetone provide the most favorable conditions for ^3^MMLCT emission and afford the highest quantum yields (Φ = 0.50 and 0.38, respectively). In contrast, toluene results in a very low emission efficiency, indicating that nonradiative decay pathways far outweigh radiative processes, which we tentatively attribute to π-interaction effects on both the open and closed conformations.

**Figure 7:**
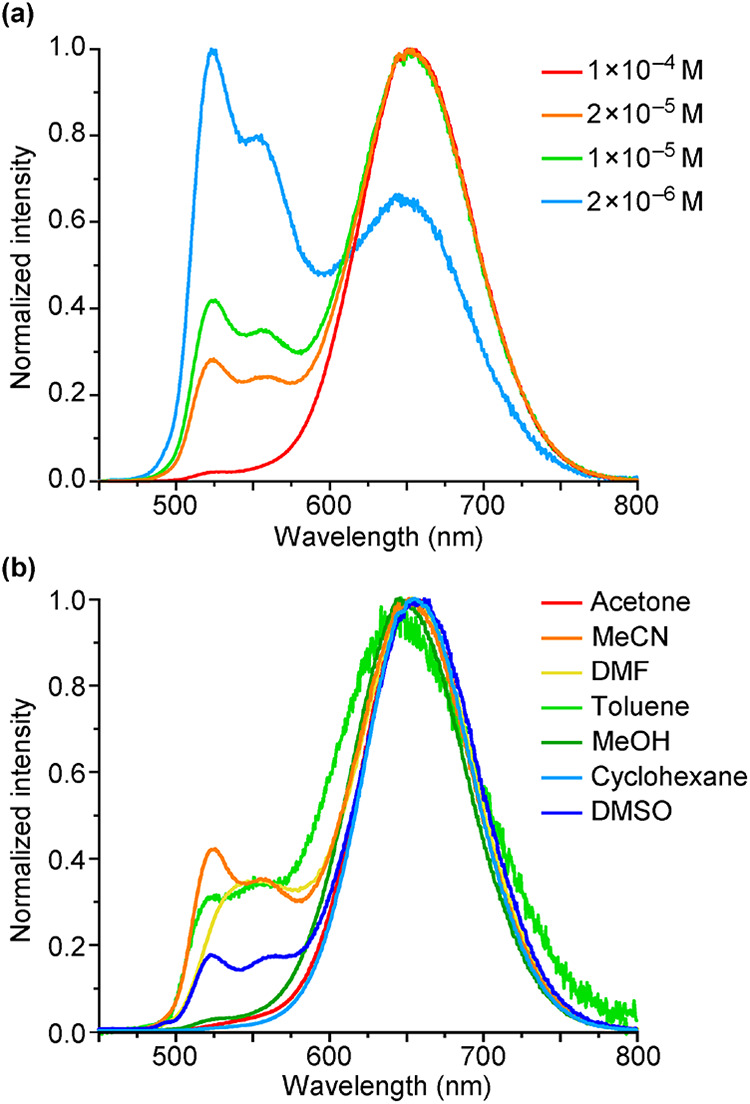
(a) Emission spectra of complex **2** at different concentrations at 298 K (λ_exc_ = 430 nm). (b) Emission spectra of complex **2** in different solvents at 298 K (1.0 × 10^–5^ M; λ_exc_ = 430 nm).

**Table 3:** Emission Data of Complex **2** in Different Solvents at 298 K^a^

**medium**	**λ**_**em**_ ^b^ **(nm)**	**Φ** ^c^
acetone	540, *657*	0.38
MeCN	525, 556, *655*	0.28
DMF	550, *655*	0.30
toluene	522, 553, *640*	0.01
MeOH	526, *648*	0.24
cyclohexane	655	0.50
DMSO	524, 563, *655*	0.05

a Excitation wavelength was 430 nm for all measurements.

b The most intense peak is italicized.

c Emission quantum yield.

The effect of temperature on the equilibrium between the open and closed conformations of complex **2** was also investigated by recording the emission spectra in DMF solution (1.0 × 10^–5^ M) over the 220–309 K range. Figure 8 presents the spectra normalized at the maximum of the low-energy band to facilitate comparison of the relative intensities of the high- and low-energy bands. A comparison based on the absolute emission intensities is provided in Figure S33 and Table S5. Upon decreasing the temperature from 309 to 270 K, the relative intensity of the low-energy band with respect to the high-energy band increases markedly, whereas it remains essentially constant between 270 and 220 K. Although the emissions arising from the open and closed conformations may be enhanced to different extents as a result of reduced nonradiative decay rates at lower temperatures, the observed trend is consistent with a shift of the equilibrium toward the closed conformation upon cooling, in agreement with the variable-temperature absorption data.

**Figure 8:**
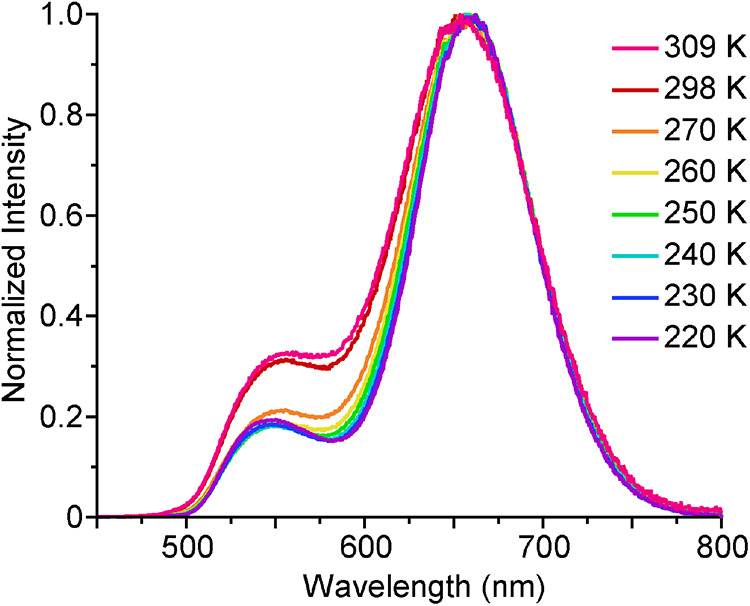
Emission spectra of complex **2** in DMF (1 × 10^–5^ M) at different temperatures.

A number of previously reported dinuclear Pt­(II) systems with flexible linkers have been shown to display equilibria involving intramolecular association, which can be modulated by temperature or by the composition of solvent mixtures.^17,26,58,70,71^ However, evidence for equilibria between two distinct emissive conformations of diphosphine-bridged systems in solution remains sparse and poorly defined. Of particular relevance to the present work, derivatives of the type [{Pt­(R-C^∧^N^∧^C)}_2_(μ-dppm)], where R-C^∧^N^∧^C = dimetalated 2,6-di­(2-naphthyl)-4-R-pyridine, can adopt two different conformations in the crystalline state, one of which features a Pt···Pt interaction, but distinct emissions arising from these forms in solution were not demonstrated.^40^ The dual emission observed for [{Pt­(C^∧^N^∧^N)}_2_(μ-dppm)]­(ClO_4_)_2_, with C^∧^N^∧^N = 4,6-diphenyl-2,2′-bipyridine, was tentatively attributed to the presence of different conformers at low concentrations, although studies on solvent and temperature dependence were not provided.^63^ An analogous complex containing C^∧^N^∧^N = 2-phenyl-6-(1H-pyrazol-3-yl)­pyridine adopts a closed conformation displaying a Pt···Pt contact and requires deprotonation to access the corresponding open form.^57^ Closely related systems of the type [{Pt­(C^∧^N^∧^N)}_2_(μ-P^∧^P)]­(PF_6_)_2_, with C^∧^N^∧^N = 4-tolyl-6-phenyl-2,2′-bipyridine and P^∧^P = dppm, dppe, or dppp, were recently reported to display dual emission only from the dppe derivative, which was attributed to thermally equilibrated triplet excited states rather than to conformational equilibria.^33^ In this context, complex **2** constitutes a distinctive case in which an equilibrium between two different emissive conformations can be conclusively demonstrated spectroscopically in solution at room temperature, which does not require any chemical transformation.

## Conclusions

In summary, this work demonstrates how diphosphine topology controls intramolecular association in dinuclear Pt­(II) complexes and highlights the key role of the dimetalated N^∧^C^∧^C ligand in enabling efficient emission and dynamic aggregation behavior. In particular, the dppm-bridged derivative **2** establishes in solution a dynamic equilibrium between an open conformation and a Pt···Pt-associated closed form, giving rise to distinct emissive excited states. The closed form features a metallophilic Pt···Pt interaction and produces a ^3^MMLCT emission, whereas the open form lacks such interaction and displays ^3^ILCT/MLCT emission from the individual {Pt­(dmtppy)} subunits. This equilibrium is solvent- and temperature-dependent, providing a rare example of reversible intramolecular metallophilic association in a dinuclear dppm-bridged Pt­(II) complex. Complexes **3**–**7** display significant emission in solution and in polystyrene matrices arising from ^3^ILCT/MLCT excited states localized on the {Pt­(dmtppy)} fragments, consistent with the absence of intramolecular metallophilic assembly. Notably, their quantum yields in fluid solution depend strongly on linker length and structure, which can be rationalized in terms of the ability of the linker to restrict or facilitate nonradiative deactivation pathways, particularly intramolecular triplet–triplet annihilation. In the case of complex **3**, intermolecular π-stacking interactions between the aromatic rings of the dmtppy ligand are responsible for the observation of dual emission in the solid state.

These findings highlight the potential of diphosphine-bridged architectures as dynamic luminescent systems in which intramolecular aggregation and excited-state properties can be modulated through bridge design and external stimuli, offering new opportunities for the development of responsive platinum-based emitters.

## Experimental Section

### General Considerations, Materials, and Instrumentation

Synthesis-grade solvents and diphosphines were used as received from commercial suppliers. The preparation of the complex Bu_4_N­[Pt­(dmtppy)­Cl] followed the previously established method.^64^ NMR characterization was performed at 298 K using a 400 or 600 MHz Bruker Avance spectrometer. Chemical shifts for ^1^H and ^13^C nuclei are reported in ppm relative to tetramethylsilane, calibrated via the residual signals of nondeuterated solvents. External standards of H_3_PO_4_ and Na_2_PtCl_6_ in water were employed for ^31^P and ^195^Pt NMR referencing, respectively. Elemental analysis was conducted by using a LECO CHNS-932 microanalyzer. High-resolution mass spectra (ESI-HRMS) were obtained on an Agilent 6220 Accurate-Mass TOF LC/MS. Details regarding X-ray diffraction and photophysical instrumentation are provided in the Supporting Information.

### General Procedure for the Synthesis of Dimers [{Pt­(dmtppy)}_2_(μ-P^∧^P)] (**2–8**)

To a solution of Bu_4_N­[Pt­(dmtppy)­Cl] (100 mg, 0.124 mmol) in CH_2_Cl_2_ (10 mL) was added the appropriate diphosphine (0.068 mmol). The mixture was stirred for 30 min and then passed through a short silica gel chromatography column using CH_2_Cl_2_ as the eluent. The yellow-orange fraction was collected and solvent evaporation yielded complexes **2–8**.

#### [{Pt­(dmtppy)}_2_(μ-dppm)] (**2**)

Red solid. Yield: 57 mg, 63%. ^1^H NMR (600 MHz, CD_2_Cl_2_): δ 7.98 (s broad, 8H), 7.36–7.27 (m, 16H), 7.23 (ddd, *J*_H–H_ = 7.9, 7.2, 1.6 Hz, 2H), 7.18–7.13 (m, 6H), 6.97 (s broad, 2H), 6.83 (s broad, 2H), 6.66 (dd, *J*_H–H_ = 5.6, 1.0 Hz, 2H), 6.63 (d, *J*_H–H_ = 7.5 Hz, 2H), 6.53 (s with satellites, *J*_Pt–H_ = 67 Hz, 2H), 6.41 (d broad, *J*_H–H_ = 7.3 Hz, 2H), 5.71 (ddd, *J*_H–H_ = 7.1, 5.7, 1.5 Hz, 2H), 4.39 (t, *J*_P–H_ = 10.4 Hz, 2H), 2.43 (s, 6H), 1.73 (s, 6H). ^13^C­{^1^H} APT NMR (151 MHz, CD_2_Cl_2_): δ 178.4 (d, *J*_C–P_ = 105.0 Hz, C), 167.1 (t, *J*_Pt–C_ ∼ 68 Hz, *J*_C–P_ = 3.1 Hz, C), 155.3 (t, *J*_C–P_ = 4.2 Hz, C), 152.6 (CH), 152.4 (C), 143.6 (*J*_Pt–C_ ∼ 48 Hz, C), 143.1 (C), 141.4 (t, *J*_C–P_ ∼ 3 Hz, CH), 140.9 (C), 137.1 (m, C), 137.0 (C), 136.4 (C), 136.5 (CH), 134.1 (C), 133.3 (broad s, CH), 130.2 (CH), 129.8 (CH), 128.9 (m, CH), 127.2 (CH), 123.9 (CH), 121.1 (CH), 120.6 (*J*_Pt–C_ ∼ 68 Hz, CH), 118.7 (CH), 118.6 (CH), 118.5 (CH), 21.5 (CH_3_), 21.4 (CH_3_), 19.8 (t, *J*_C–P_ = 15.2 Hz, CH_2_). ^31^P­{^1^H} NMR (243 MHz, CD_2_Cl_2_): δ 29.0 (s with satellite system). ^195^Pt­{^1^H} NMR (129 MHz, CD_2_Cl_2_): δ – 4171.5 (^1^ *J*_Pt–P_ ∼ 2076 Hz) HRMS (ESI+, *m*/*z*) calcd for C_75_H_61_N_2_P_2_Pt_2_ [M + H]^+^: 1441.3606; found: 1441.3607. Anal. calcd for C_75_H_60_N_2_P_2_Pt_2_: C, 62.49; H, 4.20; N, 1.94. Found: C, 62.45; H, 4.28; N, 1.88.

#### [{Pt­(dmtppy)}_2_(μ-dppe)] (**3**)

This complex required purification by crystallization from CH_2_Cl_2_/acetone. Yellow-orange solid. Yield: 54 mg, 60%. ^1^H NMR (600 MHz, CD_2_Cl_2_): δ 7.71 (d, *J*_H–H_ = 7.8 Hz, 2H), 7.68–7.59 (m, 14H), 7.51 (d, *J*_H–H_= 9.2 Hz, 4H), 7.41 (ddd, *J*_H–H_ = 5.6, 1.7, 0.7 Hz, 2H), 7.36–7.32 (m, 4H), 7.30–7.23 (m, 14H), 6.72 (ddd, *J*_H–H_ = 7.4, 1.6, 0.7 Hz, 2H), 6.49 (ddd, *J*_H–H_ = 7.2, 5.6, 1.4 Hz, 2H), 6.48 (s with satellites, *J*_Pt–H_ = 67 Hz, 2H), 3.34 (s broad, 4H), 2.41 (s, 6H), 1.73 (s, 6H). ^13^C­{^1^H} APT NMR (151 MHz, CD_2_Cl_2_): δ 179.1 (d, *J*_C–P_ = 101.0 Hz, C), 168.6 (*J*_Pt–C_ ∼ 51 Hz, C), 155.7 (C), 154.3 (C), 152.3 (CH), 143.8 (C), 142.0 (C), 141.5 (CH), 140.4 (C), 138.7 (C), 138.5 (CH), 137.1 (C), 136.0 (C), 134.5 (d, *J*_C–P_ = 37.5 Hz, C), 133.4 (m, CH), 130.7 (CH), 129.9 (CH), 129.3 (m, CH), 127.3 (CH), 125.0 (CH), 123.1 (CH), 120.4 (CH), 119.6 (CH), 118.9 (CH), 23.5 (m, CH_2_), 21.6 (CH_3_), 21.4 (CH_3_). ^31^P­{^1^H} NMR (243 MHz, CD_2_Cl_2_): δ 31.0 (s with satellite system). ^195^Pt­{^1^H} NMR (129 MHz, CD_2_Cl_2_): δ – 4160.5 (^1^ *J*_Pt–P_ ∼ 2070 Hz). HRMS (ESI+, *m*/*z*) calcd for C_51_H_44_NP_2_Pt [M–{Pt­(dmtppy)}+H]^+^: 927.2597; found: 927.2600. Anal. calcd for C_76_H_62_N_2_P_2_Pt_2_: C, 62.72; H, 4.29; N, 1.92. Found: C, 62.45; H, 4.38; N, 1.91.

#### [{Pt­(dmtppy)}_2_(μ-dppp)] (**4**)

Yellow solid. Yield: 51 mg, 79%. ^1^H NMR (600 MHz, CD_2_Cl_2_): δ 7.65 (d, *J*_H–H_= 7.7 Hz, 2H), 7.63–7.59 (m, 12H), 7.57 (ddd, *J*_H–H_ = 8.0, 7.4, 1.7 Hz, 2H), 7.53 (t, *J*_H–H_ = 1.8 Hz, 2H), 7.46 (t, *J*_H–H_= 1.6 Hz, 2H), 7.31–7.25 (m, 10H), 7.21–7.15 (m, 10H), 6.75 (ddd, *J*_H–H_ = 7.7, 1.7, 0.7 Hz, 2H), 6.54 (s with satellites, *J*_Pt–H_ = 68 Hz, 2H), 6.42 (ddd, *J*_H–H_ = 7.3, 5.7, 1.6 Hz, 2H), 3.17 (dd, *J*_H–H_ = 15.4, 8.2 Hz, 4H), 2.63–2.54 (m, 2H), 2.42 (s, 6H), 1.86 (s, 6H). ^13^C­{^1^H} APT NMR (151 MHz, CD_2_Cl_2_): δ 178.9 (d, *J*_C–P_ = 99.6 Hz, C), 168.6 (d, *J*_C–P_ = 7.1 Hz, C), 155.6 (d, *J*_C–P_ = 8.0 Hz, C), 154.3 (*J*_Pt–C_ ∼ 160 Hz, C), 152.4 (CH), 143.7 (C), 142.3 (d, *J*_C–P_ = 3.4 Hz, C), 141.2 (d, *J*_Pt–C_ ∼ 50 Hz, *J*_C–P_ = 6.0 Hz, CH), 140.5 (C), 138.4 (C), 138.3 (CH), 137.0 (C), 135.9 (*J*_Pt–C_ ∼ 70 Hz, C), 135.1 (d, *J*_C–P_ = 37.7 Hz, C), 133.5 (d, *J*_C–P_ = 11.7 Hz, CH), 130.4 (CH), 130.0 (CH), 129.0 (d, *J*_C–P_ = 9.3 Hz, CH), 127.3 (CH), 124.9 (CH), 122.7 (CH), 120.3 (*J*_Pt–C_ ∼ 71 Hz, CH), 119.4 (d, *J*_C–P_ = 4.9 Hz, CH), 119.3 (CH), 118.8 (d, *J*_C–P_ = 5.2 Hz, CH), 29.0 (dd, *J*_C–P_ = 26.7, 12.8 Hz, CH_2_) 23.0 (m, CH_2_), 21.6 (CH_3_), 21.4 (CH_3_). ^31^P­{^1^H} NMR (243 MHz, CD_2_Cl_2_): δ 26.8 (s with satellites, *J*_Pt–P_ = 2041 Hz). ^195^Pt­{^1^H} NMR (129 MHz, CD_2_Cl_2_): δ – 4142.3 (^1^ *J*_Pt–P_ ∼ 2041 Hz). HRMS (ESI+, *m*/*z*) calcd for C_77_H_65_N_2_P_2_Pt_2_ [M + H]^+^: 1469.3918; found: 1469.3907. Anal. calcd for C_77_H_64_N_2_P_2_Pt_2_: C, 62.94; H, 4.39; N, 1.91. Found: C, 63.14; H, 4.54; N, 1.91.

#### [{Pt­(dmtppy)}_2_(μ-dppb)] (**5**)

This complex required purification by crystallization from CH_2_Cl_2_/CH_3_OH. Yellow solid. Yield: 62 mg, 68%. ^1^H NMR (600 MHz, CD_2_Cl_2_): δ 7.76–7.66 (m, 12H), 7.61–7.59 (m, 4H), 7.53–7.50 (m, 4H), 7.40–7.30 (m, 14H), 7.30–7.27 (m, 4H), 7.25 (d, *J*_H–H_ = 7.6 Hz, 2H), 6.70 (ddd, *J*_H–H_ = 7.5, 1.8, 0.8 Hz, 2H), 6.57 (ddd, *J*_H–H_ = 7.2, 5.6, 1.5 Hz, 2H), 6.53 (s with satellites, *J*_Pt–H_ = 70 Hz, 2H), 2.63 (dd, *J*_H–H_ = 14.3, 8.5 Hz 4H), 2.41 (s, 6H), 2.20 – 2.13 (m, 4H), 1.80 (s, 6H). ^13^C­{^1^H} APT NMR (150 MHz, CD_2_Cl_2_): δ 178.9 (d, *J*_C–P_ = 100.5 Hz, C), 168.8 (d, *J*_C–P_ = 7.5 Hz, C), 155.4 (d, *J*_C–P_ = 7.9 Hz, C), 154.3 (C), 152.5 (CH), 143.7 (C), 142.0 (d, *J*_C–P_ = 3.0 Hz, C), 140.9 (d, *J*_C–P_ = 5.4 Hz, CH), 140.4 (C), 138.6 (CH), 138.5 (C), 137.1 (C), 136.0 (C), 135.3 (d, *J*_C–P_ = 37.5 Hz, C), 133.6 (d, *J*_C–P_ = 11.9 Hz, CH), 130.6 (CH), 129.9 (CH), 129.1 (d, *J*_C–P_ = 9.4 Hz, CH), 127.3 (CH), 125.0 (CH), 122.8 (CH), 120.3 (CH), 119.6 (CH), 118.9 (d, *J*_C–P_ = 4.2 Hz, CH), 28.5 (m, CH_2_), 27.2 (d, *J*_C–P_ = 25.8 Hz, CH_2_), 21.7 (CH_3_), 21.4 (CH_3_). ^31^P­{^1^H} NMR (243 MHz, CD_2_Cl_2_): δ 28.2 (s with satellites, *J*_Pt–P_ = 2077 Hz). ^195^Pt­{^1^H} NMR (129 MHz, CD_2_Cl_2_): δ – 4151.8 (^1^ *J*_Pt–P_ ∼ 2078 Hz). HRMS (ESI+, *m*/*z*) calcd for C_53_H_48_NP_2_Pt [M-{Pt­(dmtppy)}+H]^+^: 955.2910; found: 955.2920. Anal. calcd for C_78_H_66_N_2_P_2_Pt_2_: C, 63.15; H, 4.48; N, 1.89. Found: C, 63.08; H, 4.55; N, 1.90.

#### [{Pt­(dptppy)}_2_(μ-dpph)] (**6**)

This complex required purification by crystallization from CH_2_Cl_2_/CH_3_OH. Yellow solid. Yield: 49 mg, 53%. ^1^H NMR (600 MHz, CD_2_Cl_2_): δ 7.77–7.72 (m, 10H), 7.70 (ddd, *J*_H–H_ = 8.0, 7.3, 1.6 Hz, 2H), 7.60–7.57 (m, 4H), 7.51–7.48 (m. 4H), 7.42–7.34 (m, 14H), 7.29–7.26 (m, 4H), 7.22 (d, *J*_H–H_ = 7.6 Hz, 2H), 6.68 (ddd, *J*_H–H_ = 7.5, 1.8, 0.8 Hz, 2H), 6.62 (ddd, *J*_H–H_ = 7.2, 5.6, 1.5 Hz), 6.53 (s with satellites, *J*_Pt–H_ = 71 Hz, 2H), 2.63–2.58 (m, 4H), 2.41 (s, 6H), 1.85 (s broad, 10H), 1.45–1.42 (m, 4H). ^13^C­{^1^H} APT NMR (151 MHz, CD_2_Cl_2_): δ 178.9 (d, *J*_C–P_ = 100.0 Hz, C), 168.8 (d, *J*_C–P_ = 7.7 Hz, C), 155.4 (d, *J*_Pt–C_ ∼ 150 Hz, *J*_C–P_ = 8.6 Hz, C), 154.3 (C), 152.5 (CH), 143.6 (C), 142.0 (d, *J*_C–P_ = 3.3 Hz, C), 141.0 (d, *J*_Pt–C_ ∼ 52 Hz, *J*_C–P_ = 6.6 Hz, CH), 140.4 (C), 138.6 (CH), 138.5 (C), 137.0 (C), 135.9 (C), 135.5 (d, *J*_C–P_ = 36.6 Hz, C), 133.7 (d, *J*_C–P_ = 11.1 Hz, CH), 130.5 (CH), 129.9 (CH), 129.1 (d, *J*_C–P_ = 9.4 Hz, CH), 127.3 (CH), 124.9 (CH), 122.7 (CH), 120.3 (*J*_Pt–C_ = 65 Hz, CH), 119.6 (CH), 119.5 (d, *J*_C–P_ = 5.0 Hz, CH), 118.9 (d, *J*_C–P_ = 5.5 Hz, CH), 31.3 (d, *J*_C–P_ = 15.0 Hz, CH_2_), 27.4 (d, *J*_C–P_ = 26.6 Hz, CH_2_), 26.5 (d, *J*_C–P_ = 5.8 Hz, CH_2_), 21.7 (CH_3_), 21.4 (CH_3_). ^31^P­{^1^H} NMR (243 MHz, CD_2_Cl_2_): δ 28.0 (s with satellites, *J*_Pt–P_ = 2072 Hz). ^195^Pt­{^1^H} NMR (129 MHz, CD_2_Cl_2_): δ – 4152.4 (^1^ *J*_Pt–P_ ∼ 2070 Hz). HRMS (ESI+, *m*/*z*) calcd for C_80_H_71_N_2_P_2_Pt_2_ [M + H]^+^: 1511.4388; found: 1511.4377. Anal. calcd for C_80_H_70_N_2_P_2_Pt_2_: C, 63.57; H, 4.67; N, 1.85. Found: C, 63.72; H, 4.61; N, 1.85.

#### [{Pt­(dptppy)}_2_(μ-pop)] (**7**)

Yellow-orange solid. Yield: 76 mg, 81%. ^1^H NMR (600 MHz, CD_2_Cl_2_): δ 7.83–7.75 (m, 6H), 7.73–7.65 (m, 6H), 7.63–7.59 (m, 6H), 7.52 (dd, *J*_H–H_ = 2.0, 1.5 Hz, 2H), 7.47 (t, *J*_H–H_ = 1.5 Hz, 2H), 7.32 (d, *J*_H–H_ = 5.5 Hz, 2H), 7.30–7.27 (m, 4H), 7.27–7.19 (m, 12H), 7.15 (ddd, *J*_H–H_ = 7.3, 2.8, 2.0 Hz 2H), 6.98–6.94 (m, 2H), 6.93 (tt, *J*_H–H_ = 7.3, 1.3 Hz, 2H), 6.66 (ddd, *J*_H–H_ = 7.6, 1.8, 0.8 Hz, 2H), 6.53 (ddd, *J*_H–H_ = 7.2, 5.6, 1.5 Hz, 2H), 6.33 (s with satellites, *J*_Pt–H_ = 67 Hz, 2H), 6.04 (ddd, *J*_H–H_ = 8.0, 3.9, 1.4 Hz, 2H), 2.41 (s, 6H), 1.70 (s, 6H). ^13^C­{^1^H} APT NMR (151 MHz, CD_2_Cl_2_): δ 178.2 (d, *J*_C–P_ = 100.8 Hz, C), 168.4 (d, *J*_C–P_ = 7.2 Hz, C), 161.2 (d, *J*_C–P_ = 2.4 Hz, C), 155.3 (d, *J*_C–P_ = 8.4 Hz, C), 154.2 (d, *J*_C–P_ = 1.1 Hz, C), 152.1 (d, *J*_C–P_ = 1.4 Hz, CH), 143.3 (d, *J*_C–P_ = 1.1 Hz, C), 142.2 (d, *J*_C–P_ = 4.2 Hz, C), 141.4 (d, *J*_C–P_ = 6.0 Hz, CH), 140.4 (C), 138.4 (CH), 138.3 (C), 137.6 (d, *J*_C–P_ = 12.0 Hz, CH), 137.0 (C), 136.0 (d, *J*_C–P_ = 12.0 Hz, CH), 135.6 (d, *J*_C–P_ = 1.3 Hz, C), 135.4 (d, *J*_C–P_ = 12.0 Hz, CH), 132.6 (d, *J*_C–P_ = 40.6 Hz, C), 132.6 (d, *J*_C–P_ = 1.4 Hz, CH), 131.2 (d, *J*_C–P_ = 41.6 Hz, C), 130.5 (dd, *J*_C–P_ = 31.5, 2.0 Hz, CH), 129.9 (CH), 129.0 (d, *J*_C–P_ = 9.8 Hz, CH), 128.8 (dd, *J*_C–P_ = 9.9 Hz, CH), 127.2 (CH), 124.6 (CH), 123.9 (d, *J*_C–P_ = 35.1 Hz, C), 123.7 (d, *J*_C–P_ = 9.9 Hz, CH), 122.7 (CH), 122.7 (d, *J*_C–P_ ∼ 4 Hz, CH), 120.0 (CH), 119.3 (CH), 119.3 (d, *J*_C–P_ ∼ 5 Hz, CH), 118.6 (d, *J*_C–P_ = 5.5 Hz, CH), 21.5 (CH_3_), 21.4 (CH_3_). ^31^P­{^1^H} NMR (243 MHz, CD_2_Cl_2_): δ 34.8 (s with satellites, *J*_Pt–P_ = 2091 Hz). ^195^Pt­{^1^H} NMR (129 MHz, CD_2_Cl_2_): δ – 4182.5 (^1^ *J*_Pt–P_ ∼ 2090 Hz). HRMS (ESI+, *m*/*z*) calcd for C_61_H_48_NOP_2_Pt [M-{Pt­(dmtppy)}+H]^+^: 1067.2859; found: 1067.2866. Anal. calcd for C_86_H_66_N_2_OP_2_Pt_2_: C, 64.74; H, 4.17; N, 1.76. Found: C, 64.86; H, 4.40; N, 1.74.

#### [{Pt­(dptppy)}_2_(μ-dppf)] (**8**)

This complex required purification by successive crystallizations from CH_2_Cl_2_/acetone and CH_2_Cl_2_/*n*-hexane. Yield: 51 mg, 51%. ^1^H NMR (600 MHz, CD_2_Cl_2_): δ 7.88–7.82 (m, 8H), 7.77–7.74 (m, 2H), 7.73–7.68 (m, 2H), 7.58 (d, *J*_H–H_ = 8.0 Hz, 4H), 7.51–7.43 (m, 8H), 7.41–7.36 (m, 8H), 7.30 (d, *J*_H–H_ = 5.6 Hz, 2H), 7.27 (d, *J*_H–H_ = 8.4 Hz, 4H), 7.20 (d, *J*_H–H_ = 7.6 Hz, 2H), 6.65 (d, *J*_H–H_ = 7.6 Hz, 2H), 6.61 (t, *J*_H–H_ = 6.6 Hz, 2H), 6.33 (s with satellites, *J*_Pt–H_ = 68 Hz, 2H), 4.46 (s, 4H), 4.40 (s, 4H), 2.40 (s, 6H), 1.71 (s, 6H). ^31^P­{^1^H} NMR (243 MHz, CD_2_Cl_2_): δ 28.0 (s with satellites, *J*_Pt–P_ = 2071 Hz). ^13^C­{^1^H} and ^195^Pt­{^1^H} NMR spectra could not be recorded because of the poor solubility of this product. HRMS (ESI+, *m*/*z*) calcd for C_59_H_48_FeNP_2_Pt [M-{Pt­(dmtppy)}+H]^+^: 1083.2259; found: 1083.2263. Anal. calcd for C_84_H_66_FeN_2_P_2_Pt_2_: C, 62.61; H, 4.13; N, 1.74. Found: C, 62.83; H, 4.26; N, 1.72.

## Supplementary Material



## Notes

X-ray structure determinations, crystallographic data, NMR spectra, and additional figures for the photophysical study (PDF)
