# Supplementary material for: Diphosphine Bridge Control of Intramolecular Pt···Pt Association in Dinuclear Pt(II) Complexes with a Dimetalated N∧C∧C Ligand
Source: Inorg Chem. 2026 Jun 11;65(25):14183–94. doi: 10.1021/acs.inorgchem.6c01662 (PMC13321297; doi:10.1021/acs.inorgchem.6c01662)
Supplement: Supplementary file 1 [file ic6c01662_si_001.pdf]

SUPPORTING INFORMATION

Diphosphine Bridge Control of Intramolecular Pt···Pt Association in Dinuclear Pt(II) Complexes with a Dimetalated N^C^C Ligand

Salvador R. Marín-González,[†] *Dionisio Poveda,*[†] *Delia Bautista,*[‡] *Juan Gil-Rubio,*[†] *Pablo González-Herrero*[*,†] *and Ángela Vivancos*[*,†]

[†]Departamento de Química Inorgánica, Facultad de Química, Universidad de Murcia, Campus de Espinardo, 19, 30100 Murcia, Spain.

[‡]Área Científica y Técnica de Investigación, Universidad de Murcia, Campus de Espinardo, 21, 30100 Murcia, Spain.

E-Mail: pgh@um.es; angela.vivancos@um.es

Contents:

1. Photophysical characterization 2
2. NMR spectra of new compounds 3
3. Simulated $^{31}P\{^{1}H\}$ NMR spectra 23
4. X-ray structure determinations 26
5. Additional photophysical data 30
6. References 38

1. Photophysical characterization

UV-vis absorption spectra and diffuse reflectance spectra were registered on a Perkin-Elmer Lambda 750S spectrophotometer equipped with a 60 mm integrating sphere; for reflectance measurements, Nujol mulls of solid samples between polyethylene sheets were employed. Excitation and emission spectra were registered on a Jobin Yvon Fluorolog 3-22 spectrofluorometer. Emission spectra were corrected for detector sensitivity. The measurements in solution were carried out in a right-angle configuration using 10 mm quartz fluorescence cells. The emission data in polystyrene matrix were measured in a front-face configuration, using quartz slides as sample holders. Emission lifetimes longer than 10 μs were determined using an IBH FluoroHub controller in MCS mode and the Fluorolog's FL-1040 phosphorimeter pulsed xenon lamp as excitation source. Lifetimes shorter than 10 μs were determined using the IBH FluoroHub controller in TCSPC mode and a pulsed NanoLED source; the estimated uncertainty is ±10% or better. Emission quantum yields were determined using a Hamamatsu C11347 Absolute PL Quantum Yield Spectrometer; the estimated uncertainty is ±5% or better. Emission lifetimes and quantum yields were determined under rigorous exclusion of atmospheric oxygen.

2. NMR spectra of new compounds

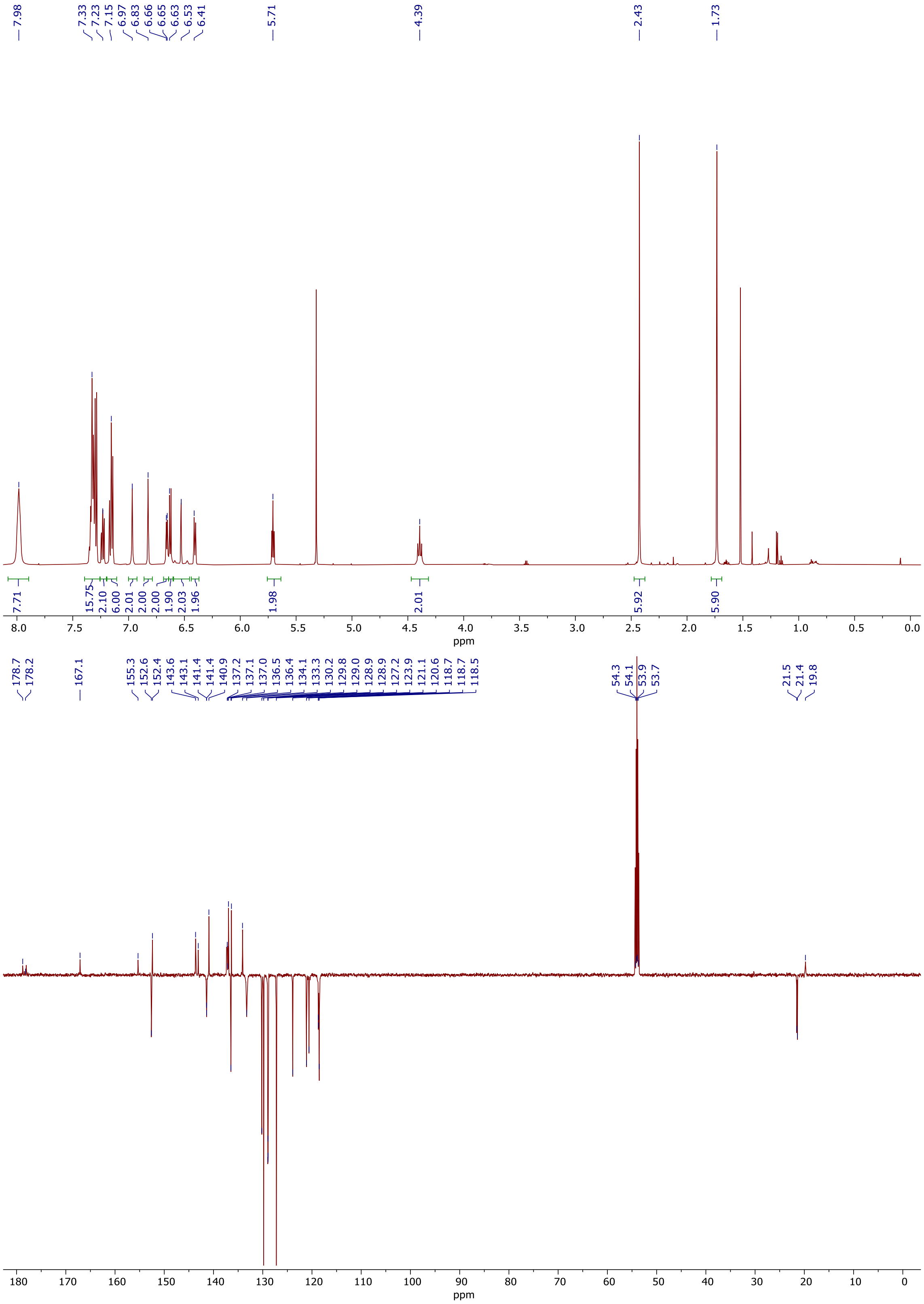

Figure S1. ^{1}H (top) and $^{13}C\{^{1}H\}$ APT (bottom) NMR spectra of [{Pt(dmtppy)}$_2${μ-dppm}] (**2**) (CD_2Cl_2, 600 and 151 MHz, respectively).

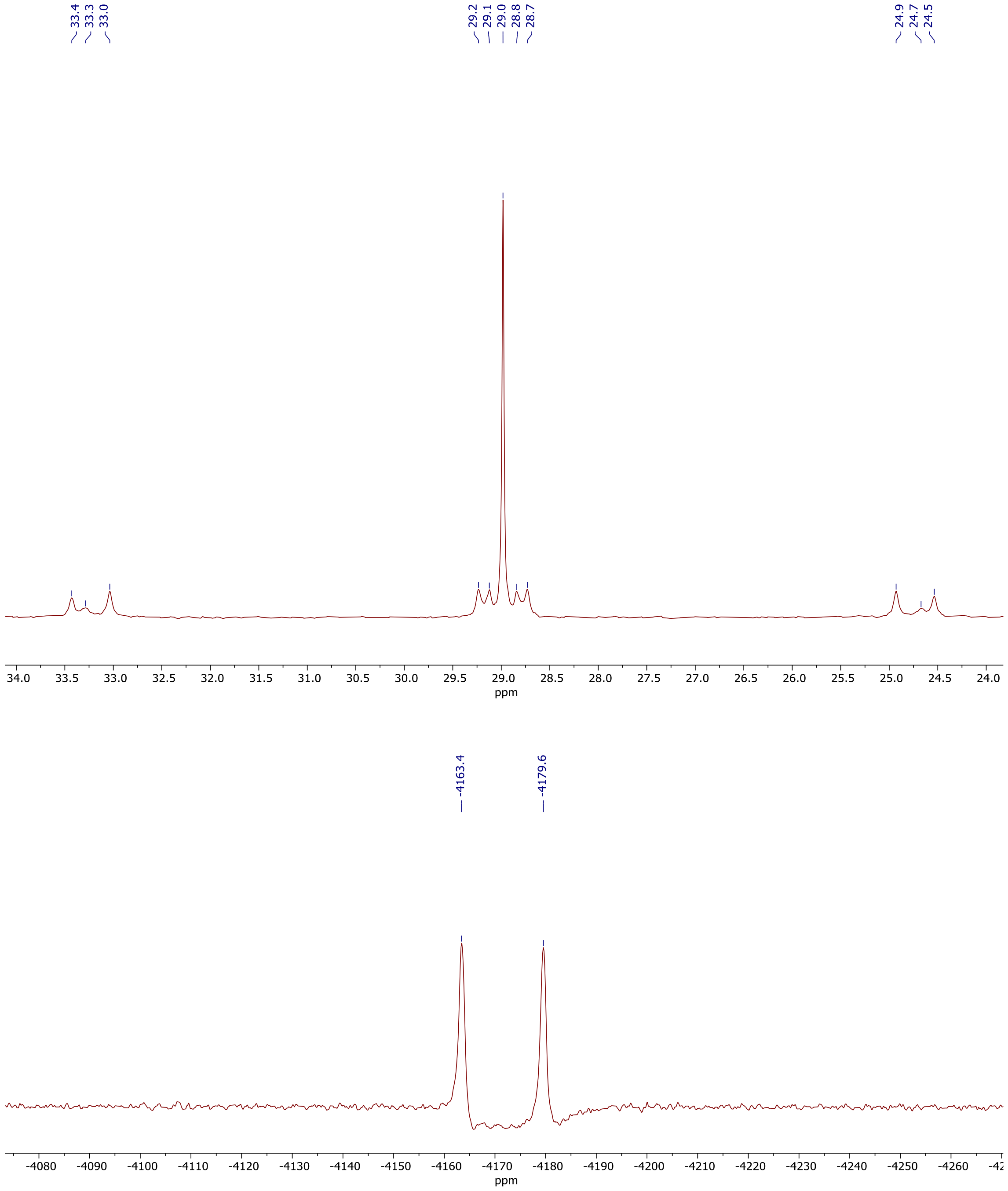

Figure S2. $^{31}P\{^{1}H\}$ (top) and $^{195}Pt\{^{1}H\}$ (bottom) NMR spectra of [{Pt(dmtppy)}$_2${μ-dppm}] (**2**) (CD_2Cl_2, 243 and 129 MHz, respectively).

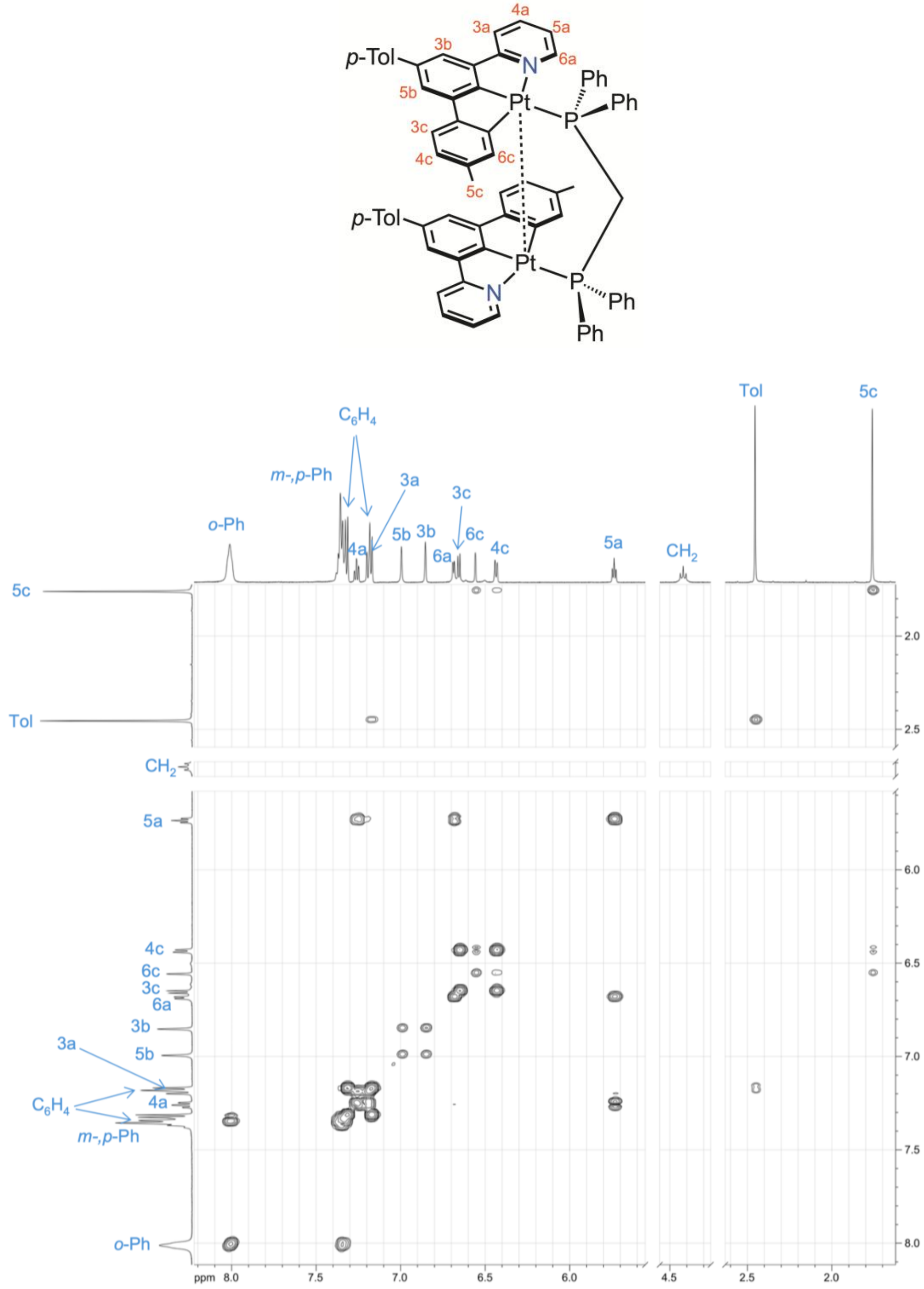

Figure S3. COSY NMR spectrum of [{Pt(dmtppy)}$_2${μ-dppm}] (**2**) (CD_2Cl_2, 600 MHz).

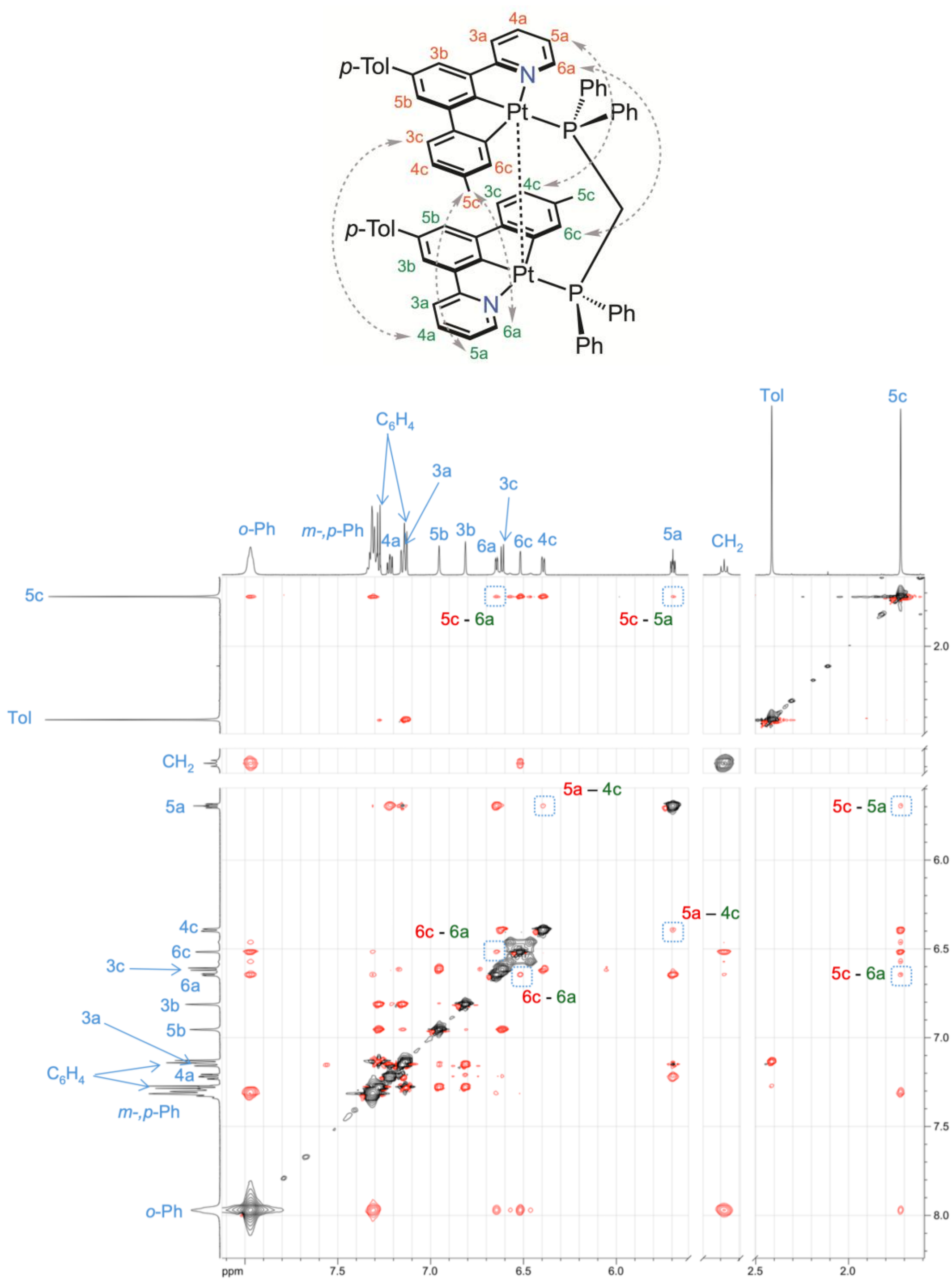

Figure S4. NOESY NMR spectrum of [{Pt(N^C^C)}$_2${μ-dppm}] (**2**) (CD_2Cl_2, 600 MHz).

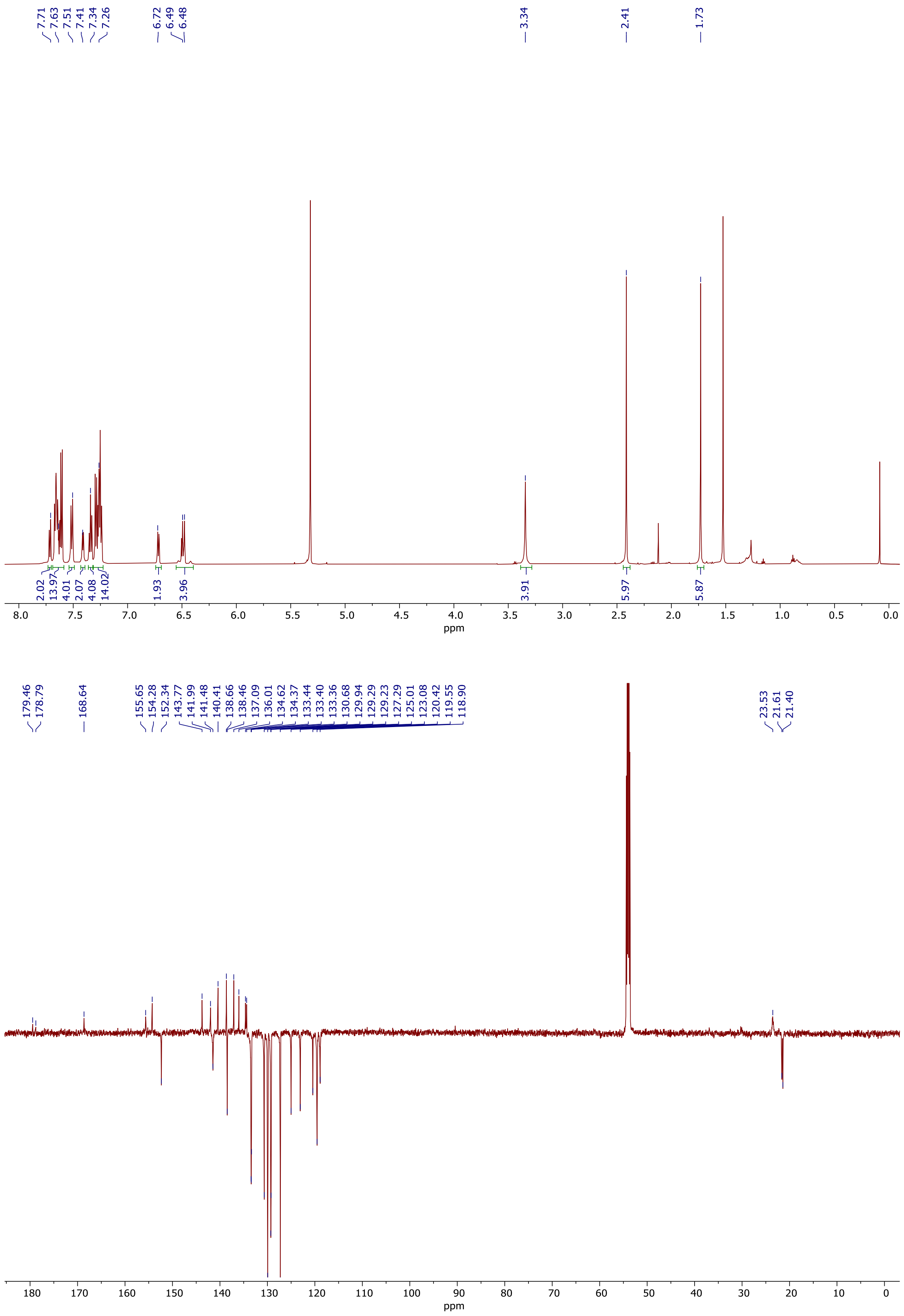

Figure S5. ^{1}H (top) and $^{13}C\{^{1}H\}$ APT (bottom) NMR spectra of [{Pt(dmtppy)}$_2${μ-dppe}] (**3**) (CD_2Cl_2, 600 and 151 MHz, respectively).

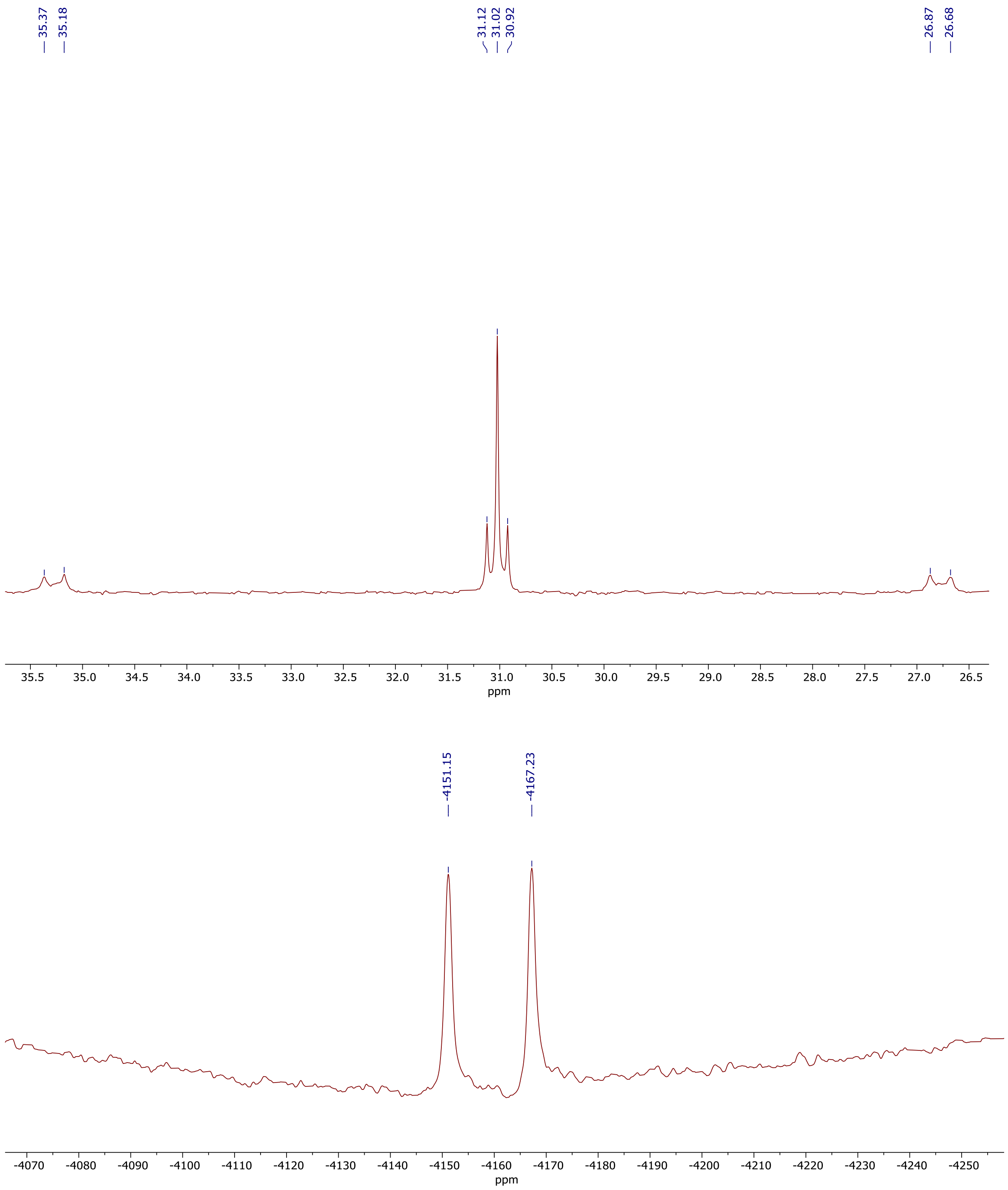

Figure S6. $^{31}P\{^1H\}$ (top) and $^{195}Pt\{^1H\}$ (bottom) NMR spectra of [{Pt(dmtppy)}$_2${μ-dppe}] (**3**) (CD_2Cl_2, 243 and 129 MHz, respectively).

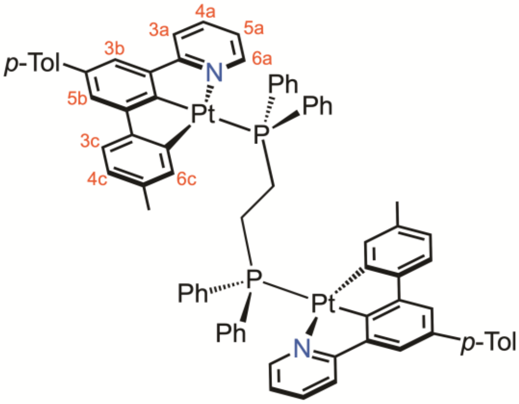

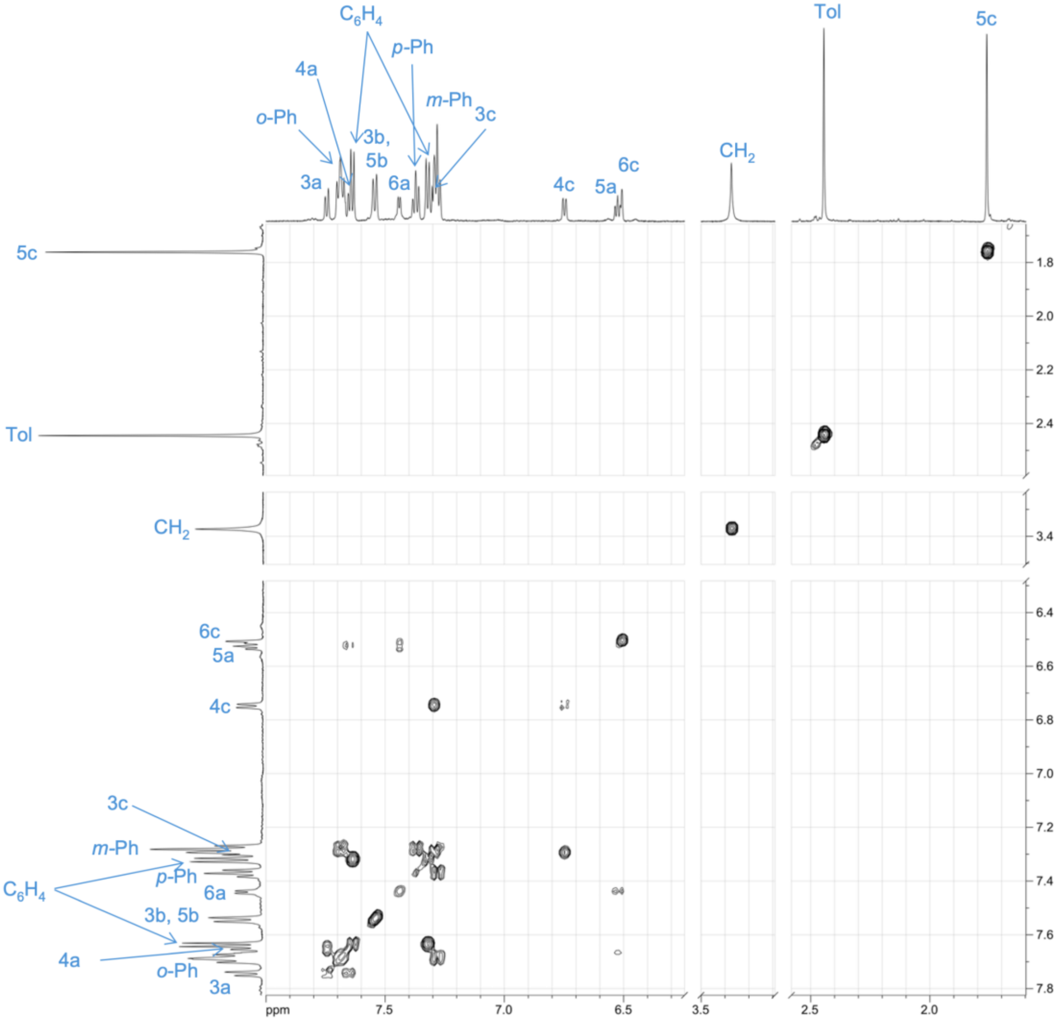

Figure S7. COSY NMR spectrum of [{Pt(dmtppy)}$_2${μ-dppe}] (**3**) (CD_2Cl_2, 600 MHz).

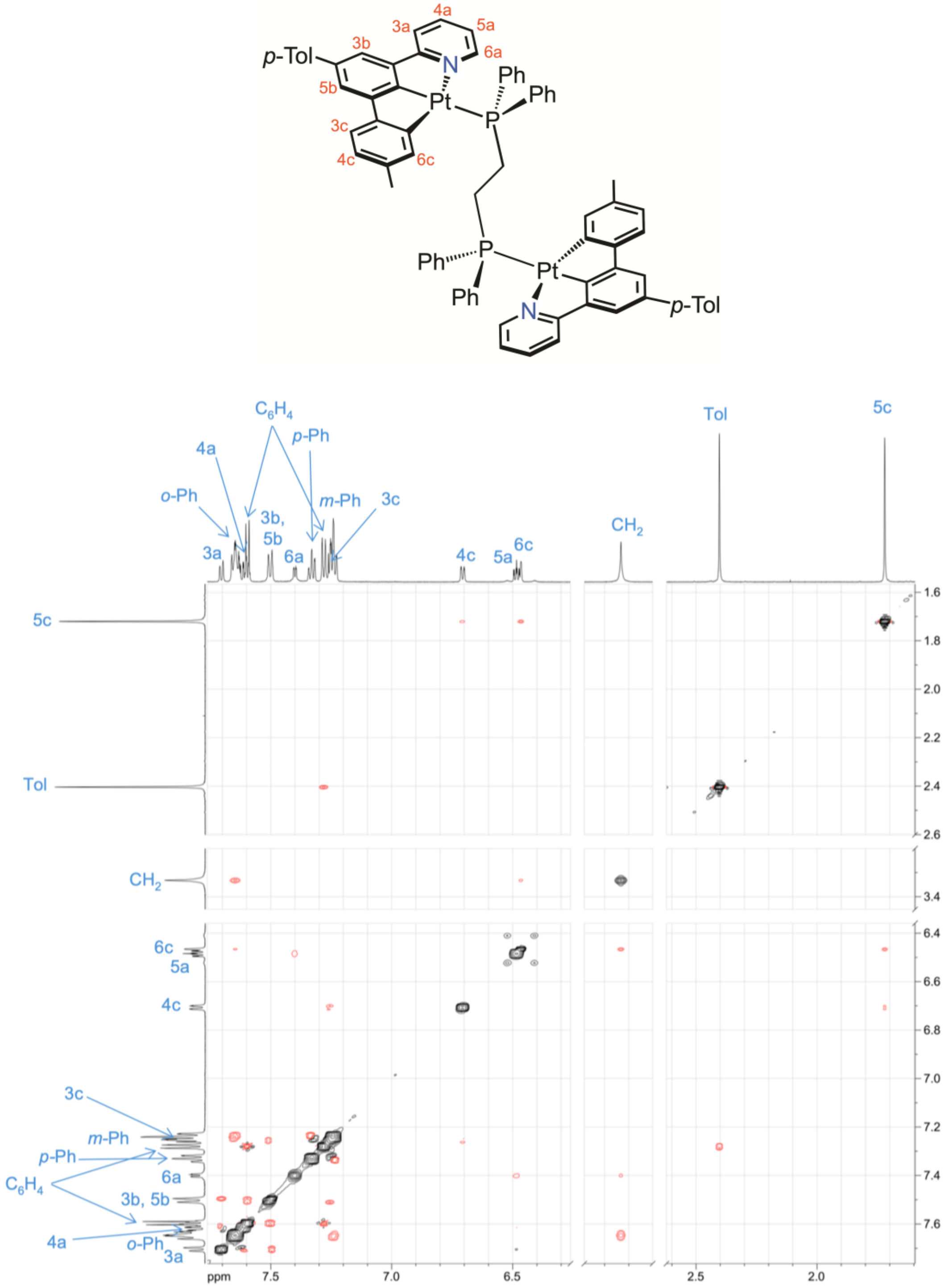

Figure S8. NOESY NMR spectrum of [{Pt(dmtppy)}$_2${μ-dppe}] (**3**) (CD_2Cl_2, 600 MHz).

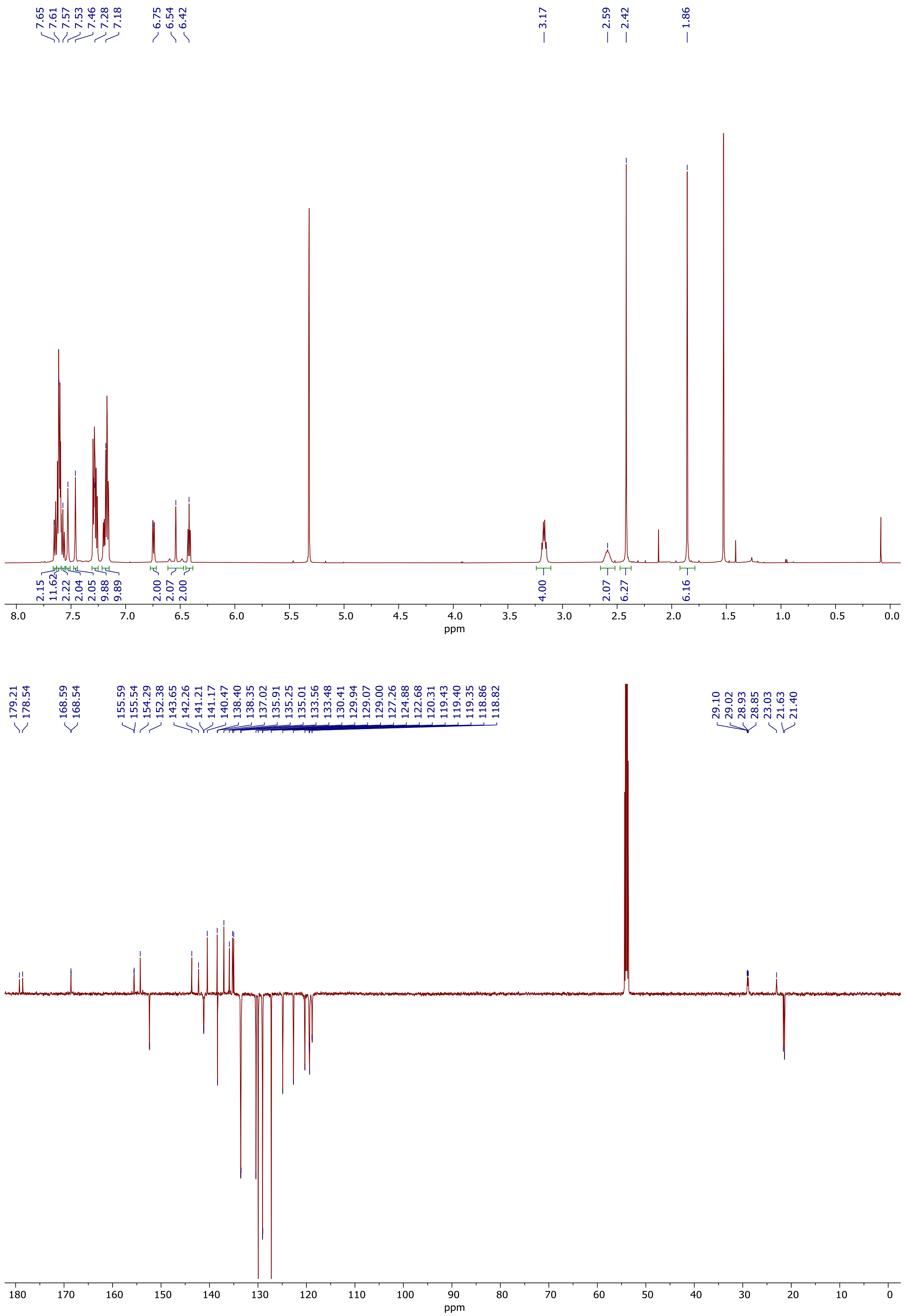

Figure S9. ^{1}H (top) and $^{13}C\{^{1}H\}$ APT (bottom) NMR spectra of [{Pt(dmtppy)}$_2${μ-dppp}] (**4**) (CD_2Cl_2, 600 and 151 MHz, respectively).

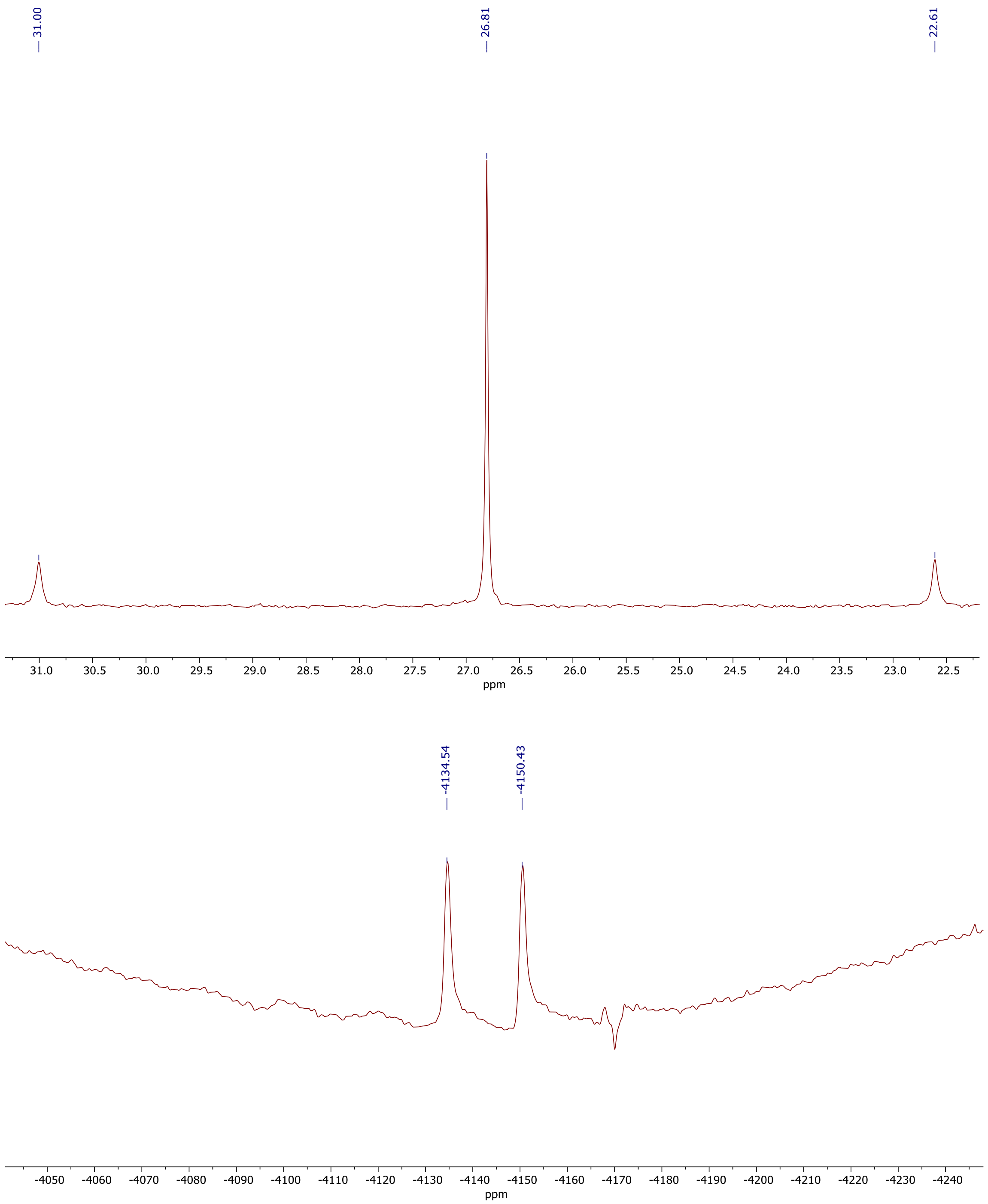

Figure S10. $^{31}P\{^1H\}$ (top) and $^{195}Pt\{^1H\}$ (bottom) NMR spectra of [{Pt(dmtppy)}$_2${μ-dppp}] (**4**) (CD_2Cl_2, 243 and 129 MHz, respectively).

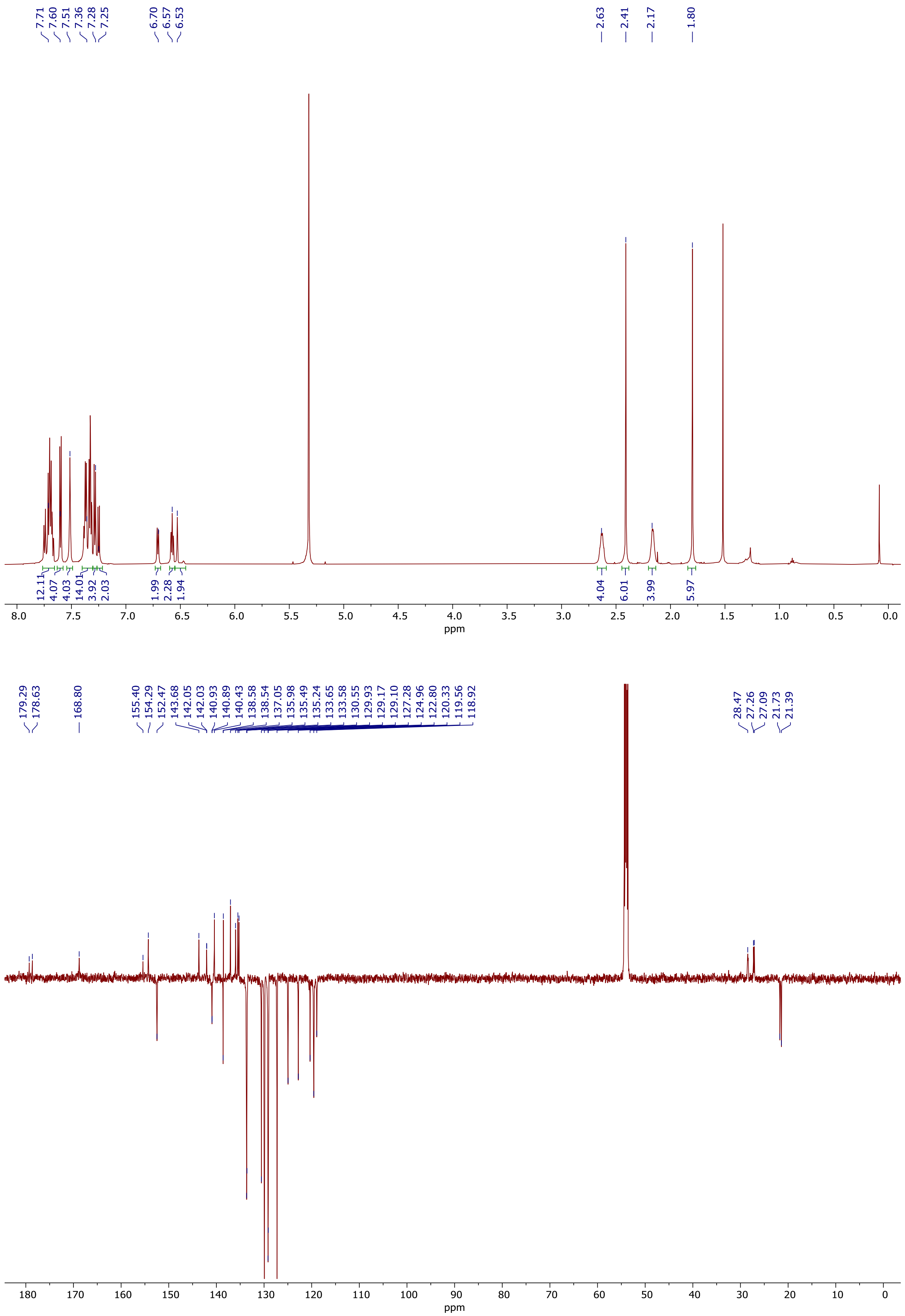

Figure S11. ^{1}H (top) and ^{13}C{^{1}H} APT (bottom) NMR spectra of [{Pt(dmtppy)}$_2${μ-dppb}] (**5**) (CD_2Cl_2, 600 and 151 MHz, respectively).

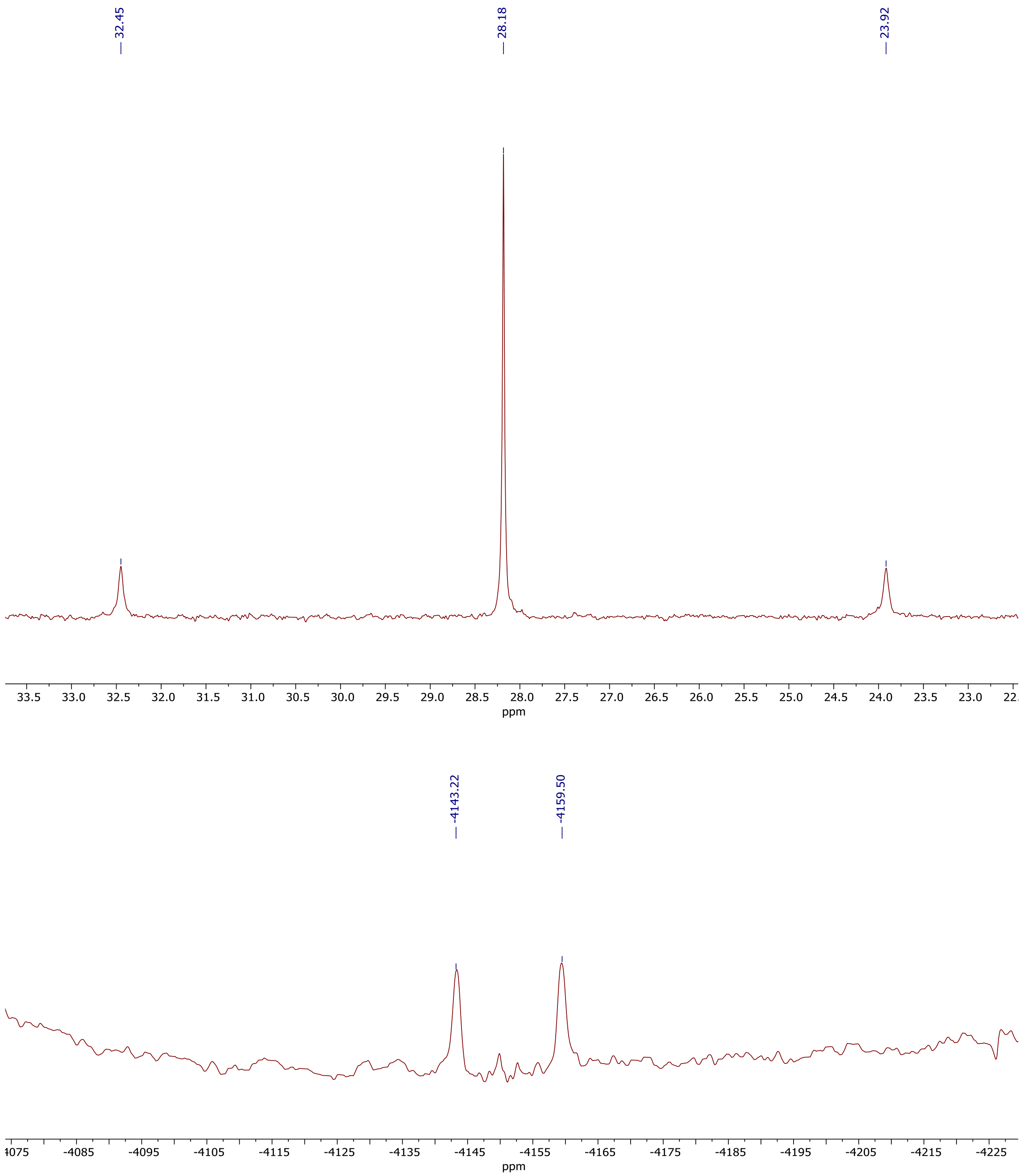

Figure S12. $^{31}P\{^{1}H\}$ (top) and $^{195}Pt\{^{1}H\}$ (bottom) NMR spectra of [{Pt(dmtppy)}$_2${μ-dpph}] (**5**) (CD_2Cl_2, 243 and 129 MHz, respectively).

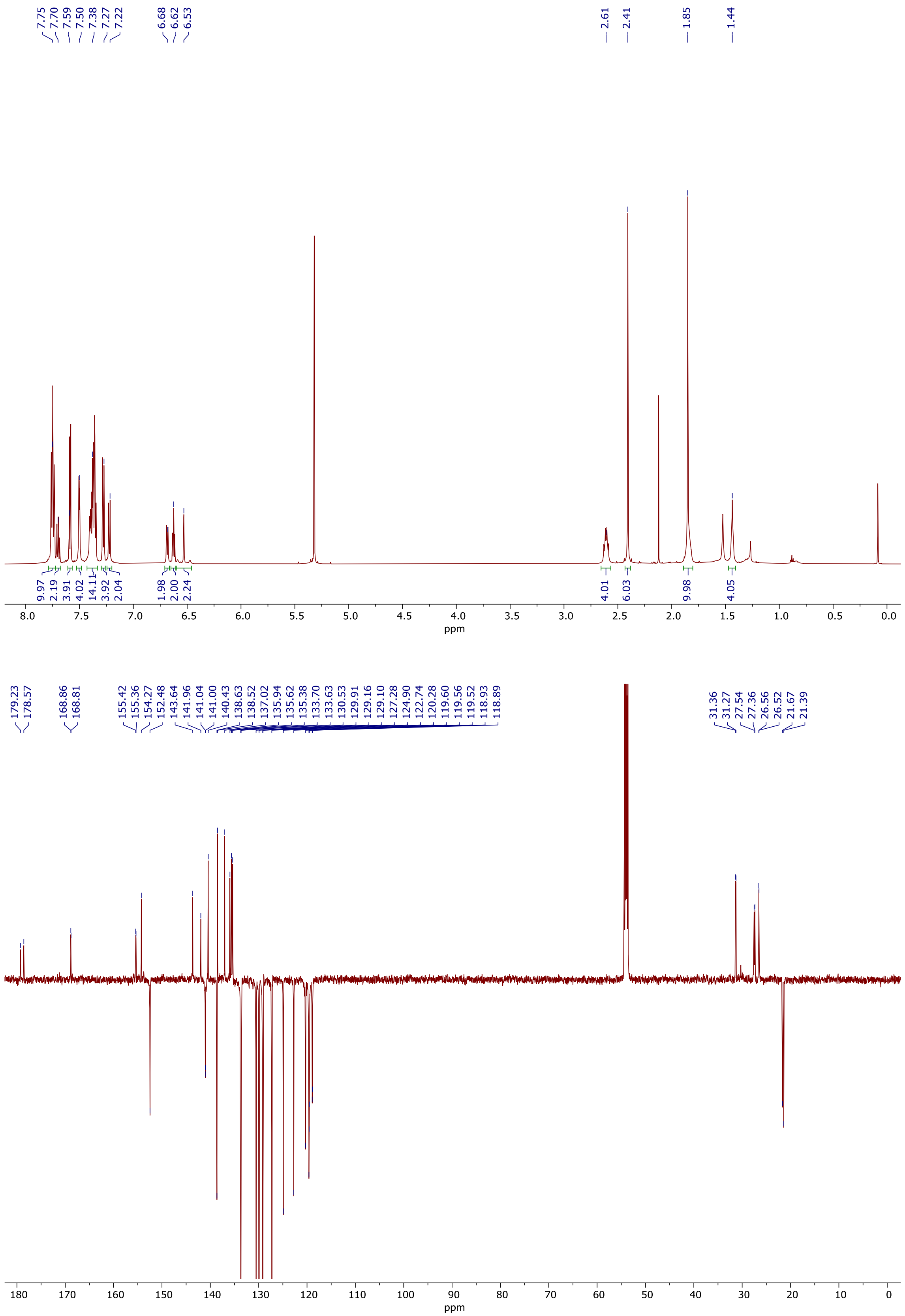

Figure S13. ^{1}H (top) and $^{13}C\{^{1}H\}$ APT (bottom) NMR spectra of [{Pt(dmtppy)}$_2${μ-dpph}] (**6**) (CD_2Cl_2, 600 and 151 MHz, respectively).

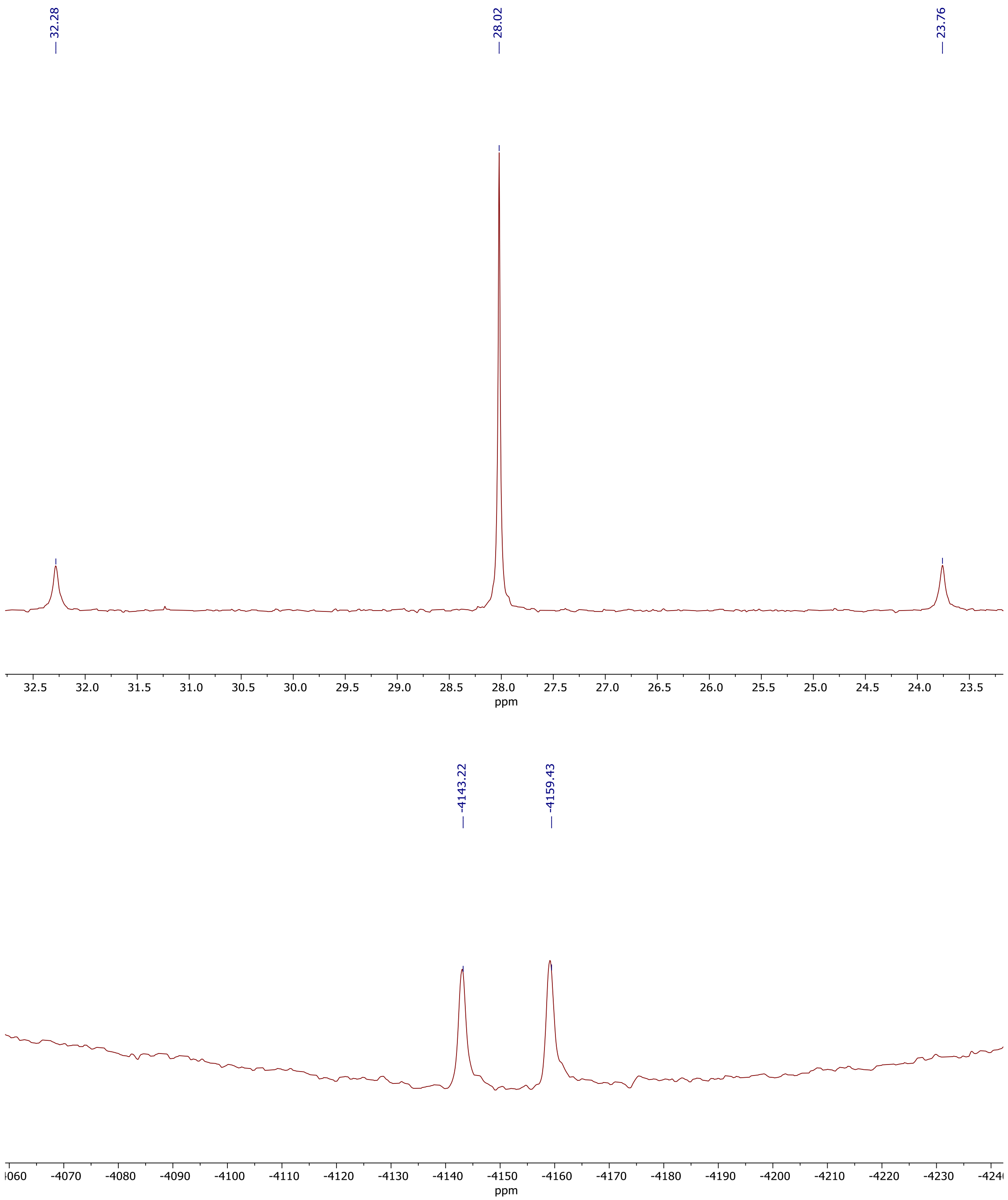

Figure S14. $^{31}P\{^1H\}$ (top) and $^{195}Pt\{^1H\}$ (bottom) NMR spectra of $[\{Pt(dmtppy)\}_2\{\mu\text{-dpph}\}]$ (**6**) (CD_2Cl_2, 243 and 129 MHz, respectively).

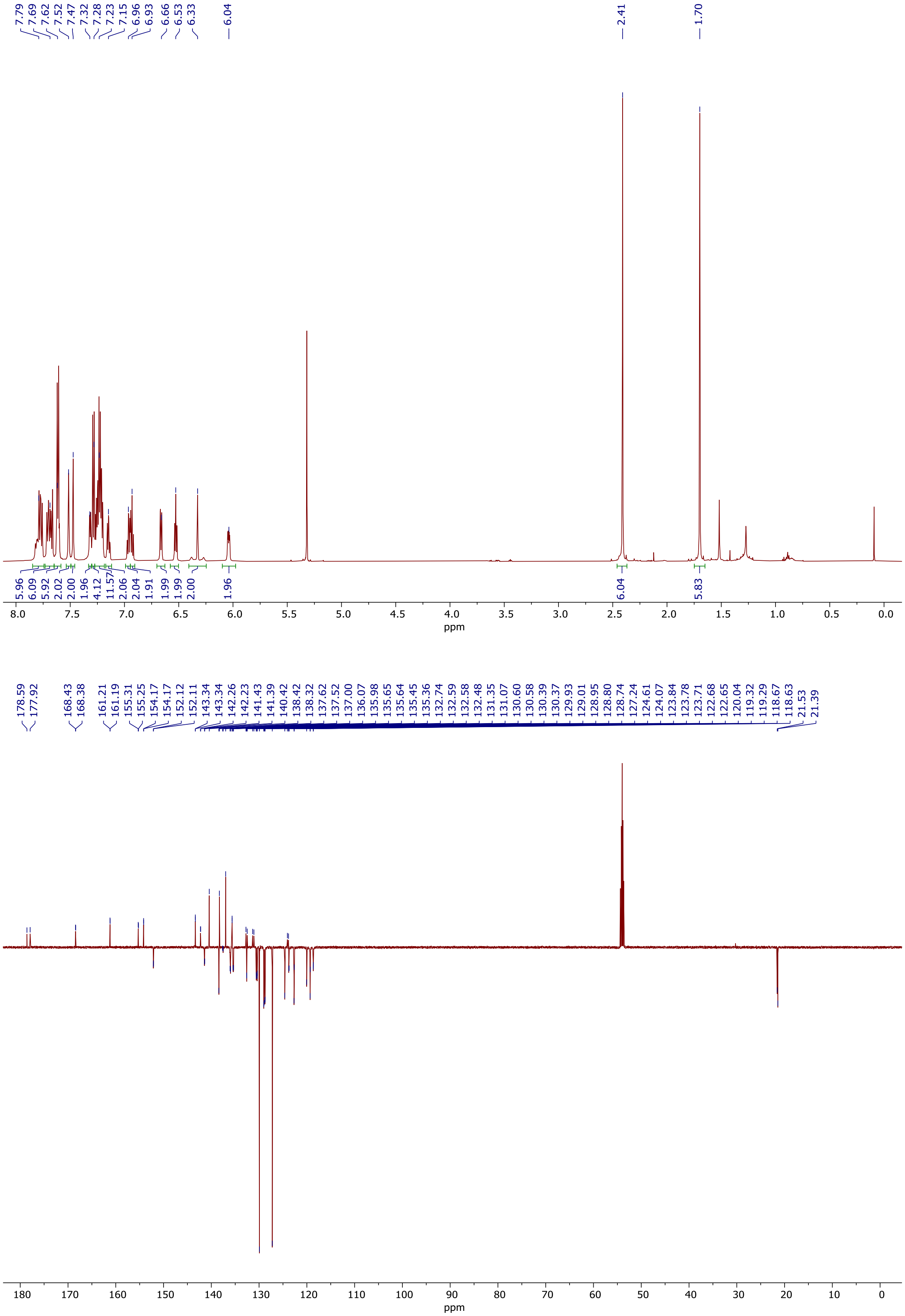

Figure S15. ^{1}H (top) and ^{13}C{^{1}H} APT (bottom) NMR spectra of [{Pt(dmtppy)}$_2${μ-pop}] (**7**) (CD_2Cl_2, 600 and 151 MHz, respectively).

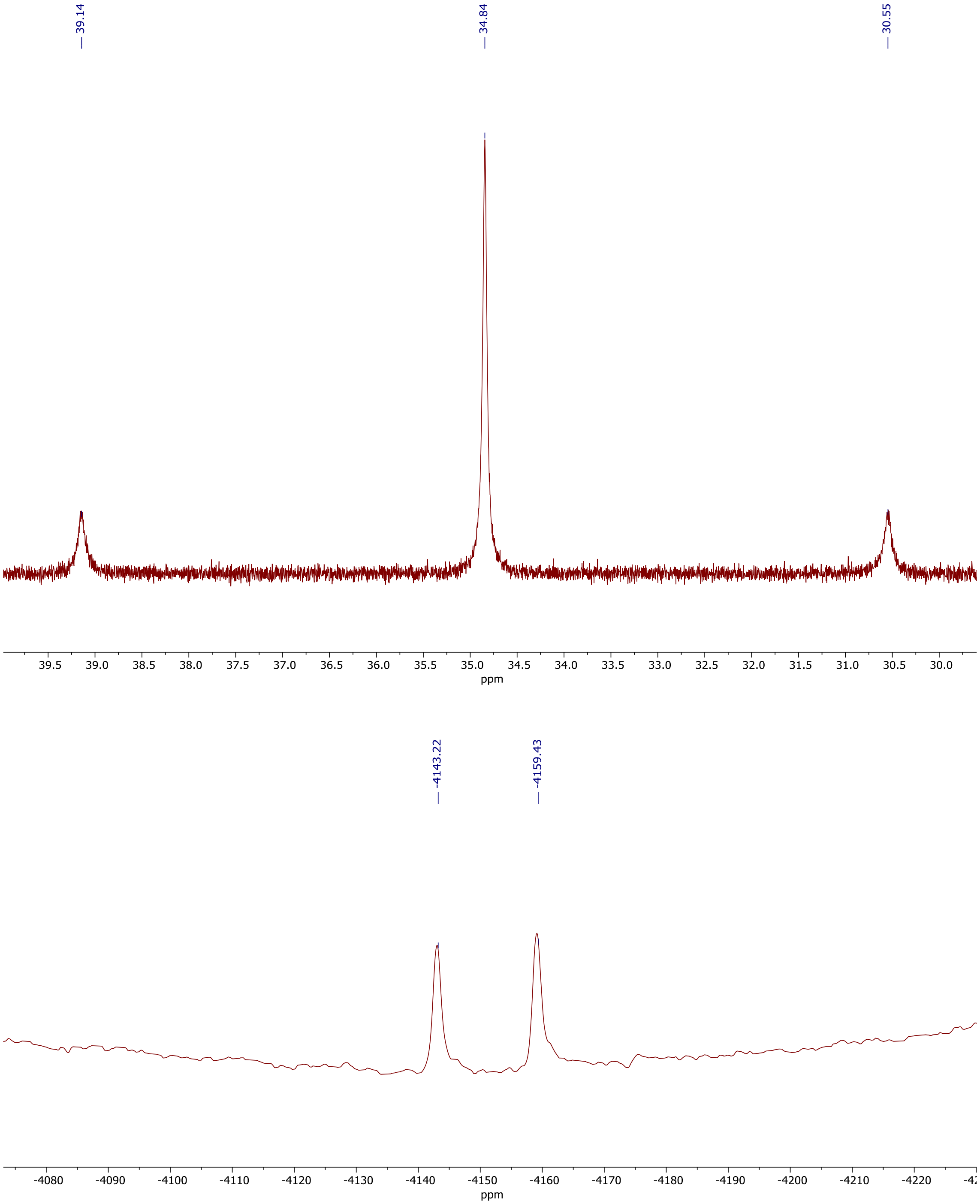

Figure S16. $^{31}P\{^{1}H\}$ (top) and $^{195}Pt\{^{1}H\}$ (bottom) NMR spectra of [{Pt(dmtppy)}$_2${μ-pop}] (**7**) (CD_2Cl_2, 243 and 129 MHz, respectively).

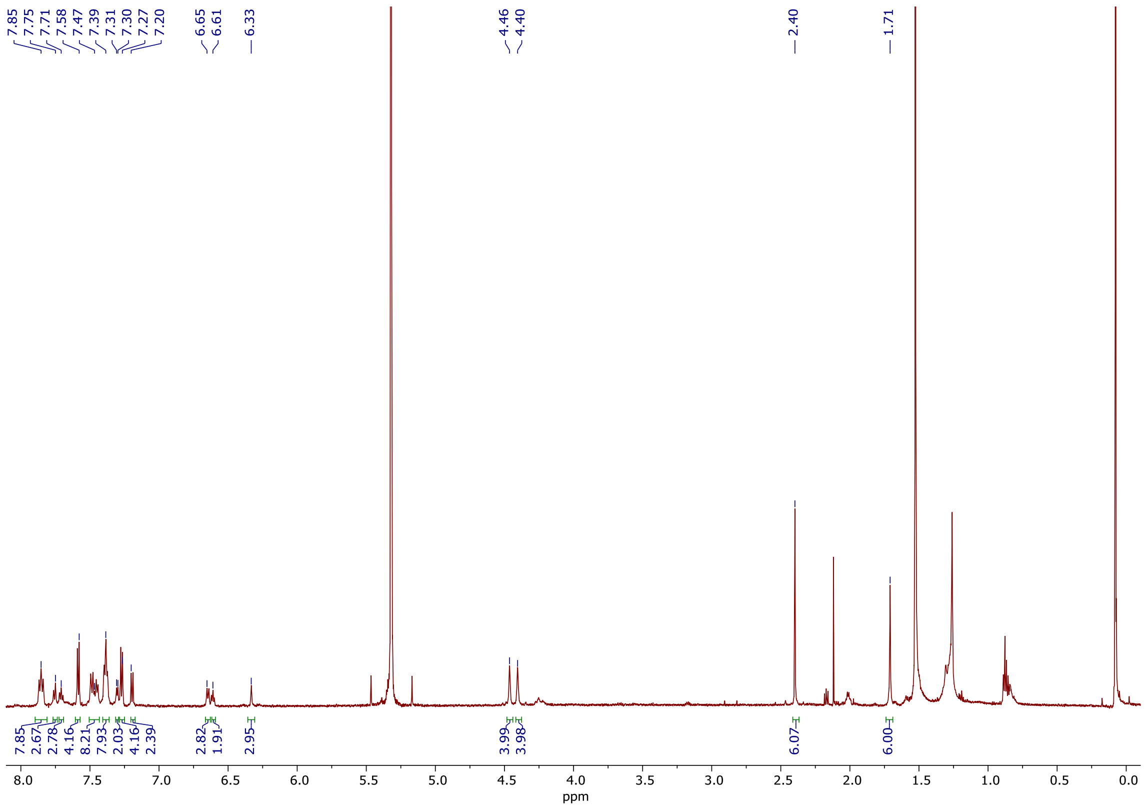

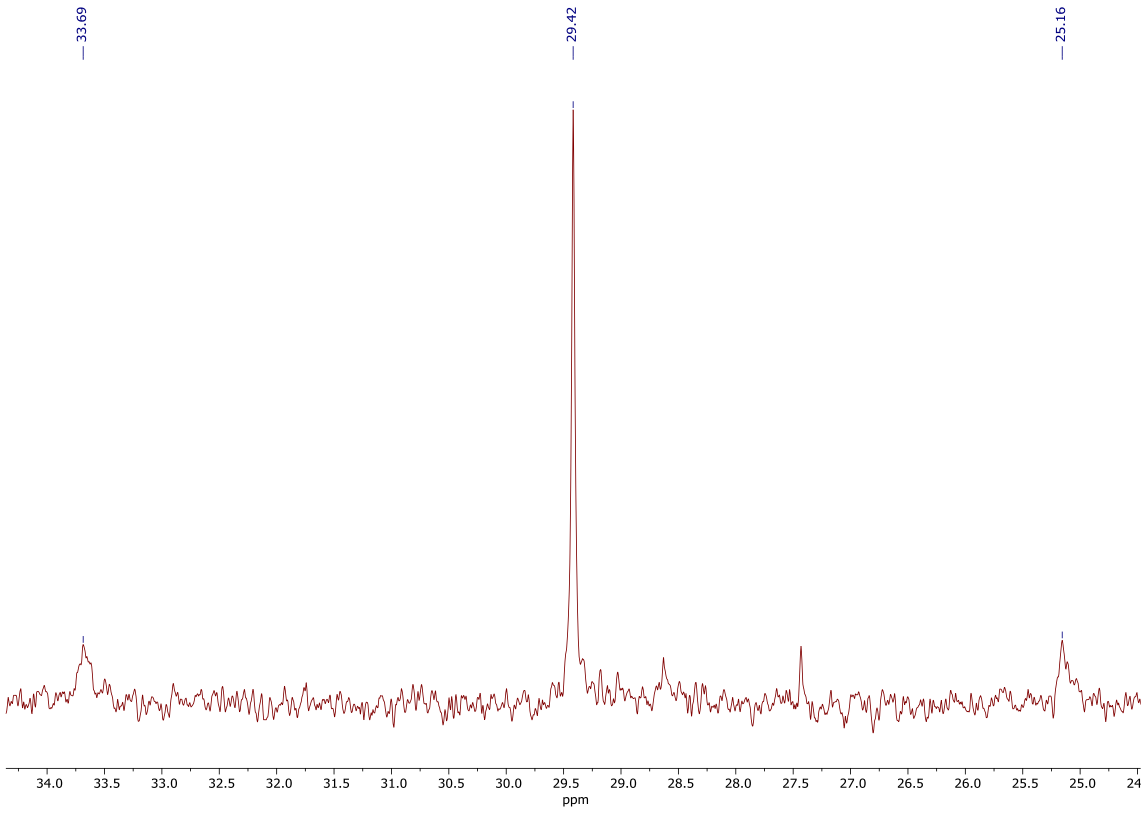

Figure S17. ^{1}H (top) and $^{31}P\{^{1}H\}$ (bottom) NMR spectra of [{Pt(dmtppy)}$_2${μ-dppf}] (**8**) (CD_2Cl_2, 600 and 243 MHz, respectively).

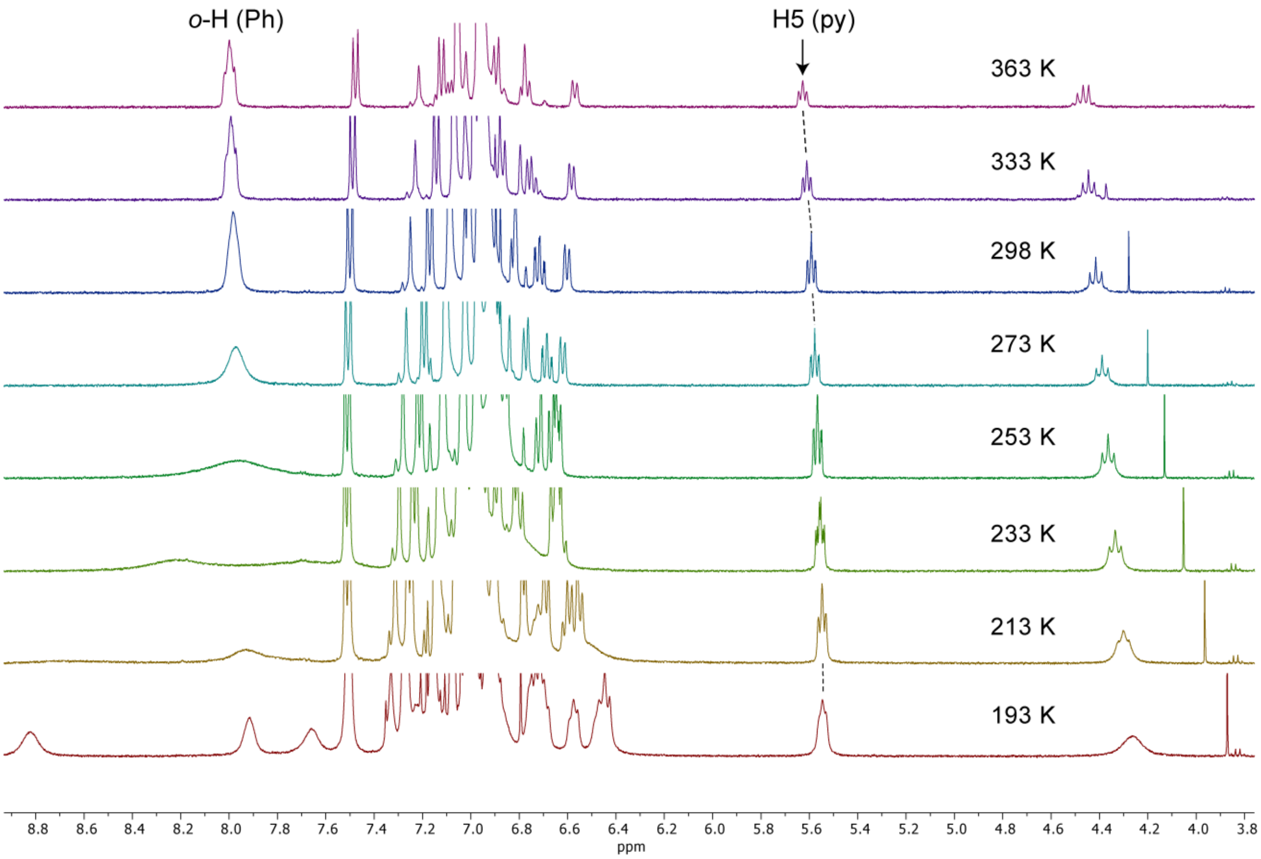

Figure S18. ^{1}H NMR spectra of **2** at different temperatures (toluene-d_8, 400 MHz).

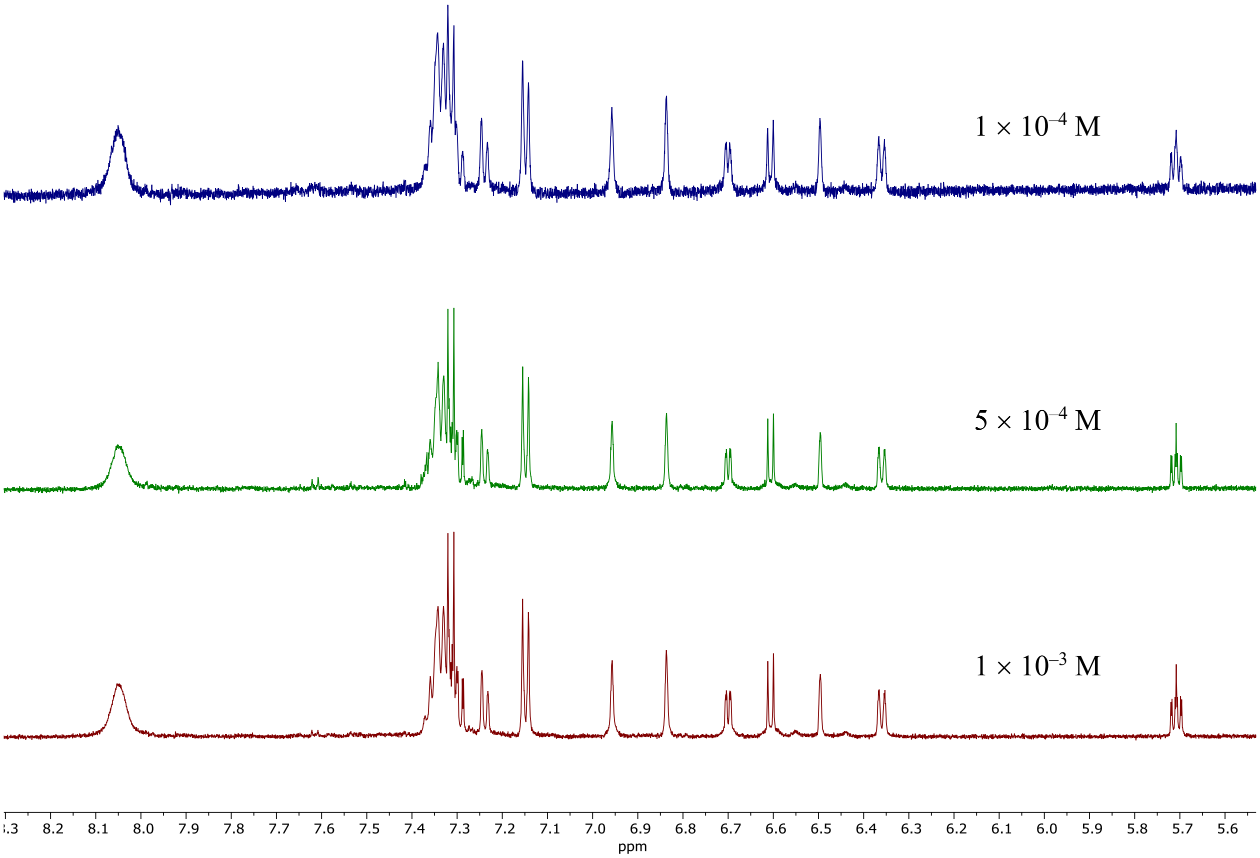

Figure S19. ^{1}H NMR spectra of **2** in CD_3CN at different concentrations (600 MHz).

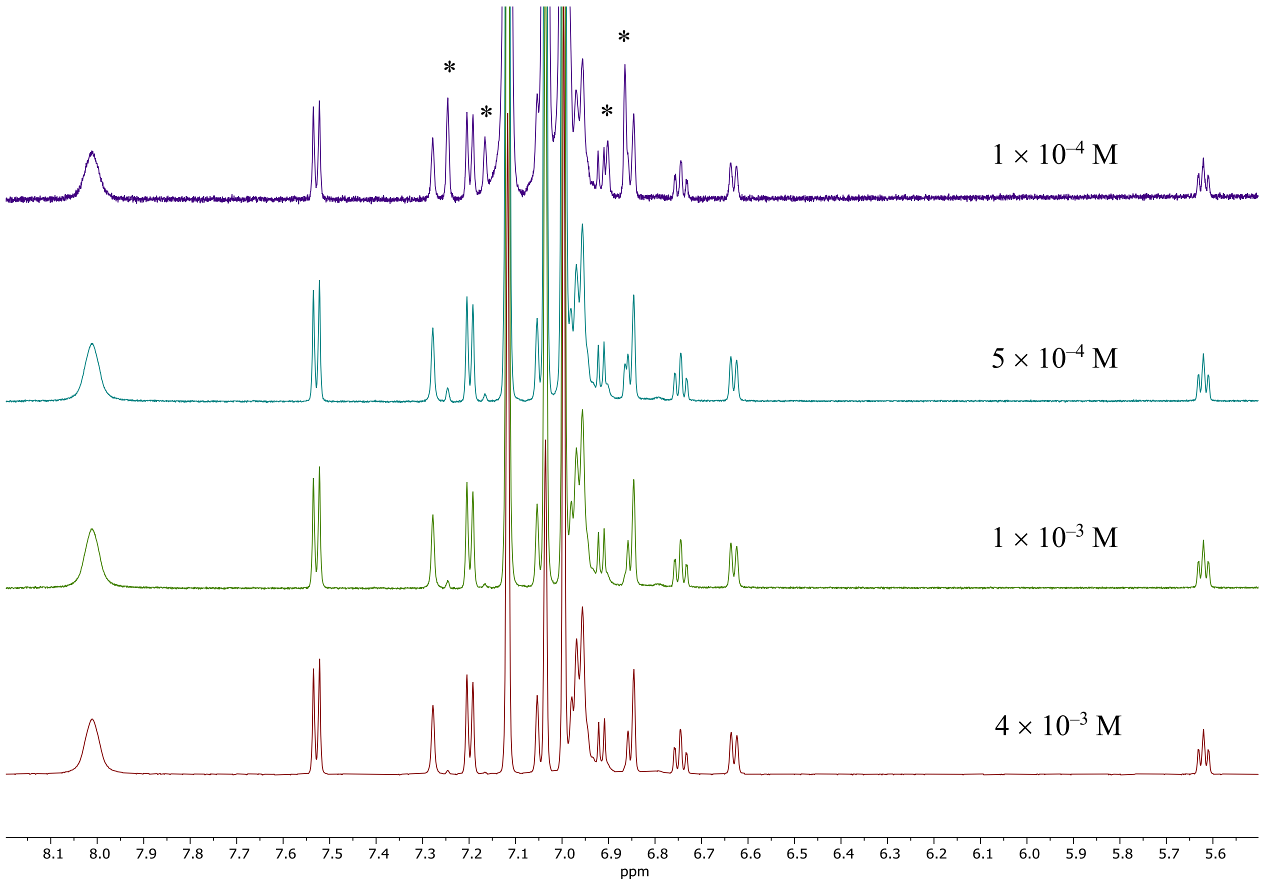

Figure S20. ^{1}H NMR spectra of **2** in toluene-d_8 at different concentrations (600 MHz). *Spin satellites of the solvent.

3. Simulated $^{31}P\{^1H\}$ NMR spectra

The $^{31}P\{^1H\}$ NMR spectrum of **2** has been simulated as the sum of the spectra of the three possible isotopologues (Figure S21). The molecules without ^{195}Pt are denoted as A_2 spin systems and give the central singlet of the spectrum. The molecules with one ^{195}Pt nuclei are $AA'X$ spin systems with two ^{195}Pt-^{31}P coupling constants: the one-bond coupling constant between ^{31}P (A) and ^{195}Pt (X) and the longer-range $A'X$ coupling constant ($^{2+3}J_{PtP}$), where the *2+3* superindex indicates that the spins A' and X interact through the P–Pt–Pt and P–C–P–Pt bonds. The sign of $^{2+3}J_{PtP}$ has been correlated with the strength of the Pt–Pt bond. Thus, if it is assumed that $^2J_{PtP}$ is negative and $^3J_{PtP}$ is positive, in compounds with a strong Pt–Pt bond the P–Pt–Pt contribution would dominate and the overall $^{2+3}J_{PtP}$ would be negative, whereas in compounds with a weak Pt···Pt interaction the P–C–P–Pt contribution would be larger and the overall $^{2+3}J_{PtP}$ value would be positive.[1] In complex **2**, the relative intensities of the lines of the $AA'X$ part of the spectrum suggest that $^1J_{PtP}$ and $^{2+3}J_{PtP}$ have the same sign. Since $^1J_{PtP}$ values are generally positive,[2] $^{2+3}J_{PtP}$ should also be positive. This means that the three-bond contribution to the coupling has a larger magnitude than the two-bond one, which is in agreement with the relatively long Pt–Pt distance observed in the crystal structure of **2**. Finally, the isotopologues with two ^{195}Pt nuclei constitute $AA'XX'$ systems. A $^1J_{PtPt}$ value of ca. 140 Hz was estimated, although an accurate fitting of the simulated spectrum to the experimental one of this part was not possible because of the broadness of the lower intensity peaks.

The $^{31}P\{^1H\}$ NMR spectrum of **3** was simulated in the same way as for **2** (Figure S22). However, in this case neither the $^{195}Pt(A)$–$^{31}P(X')$ nor the $^{195}Pt(X)$–$^{195}Pt(X')$ coupling were observed, which is in agreement with the longer bridge of the diphosphine ligand and the lack of Pt···Pt interaction in the extended conformation.

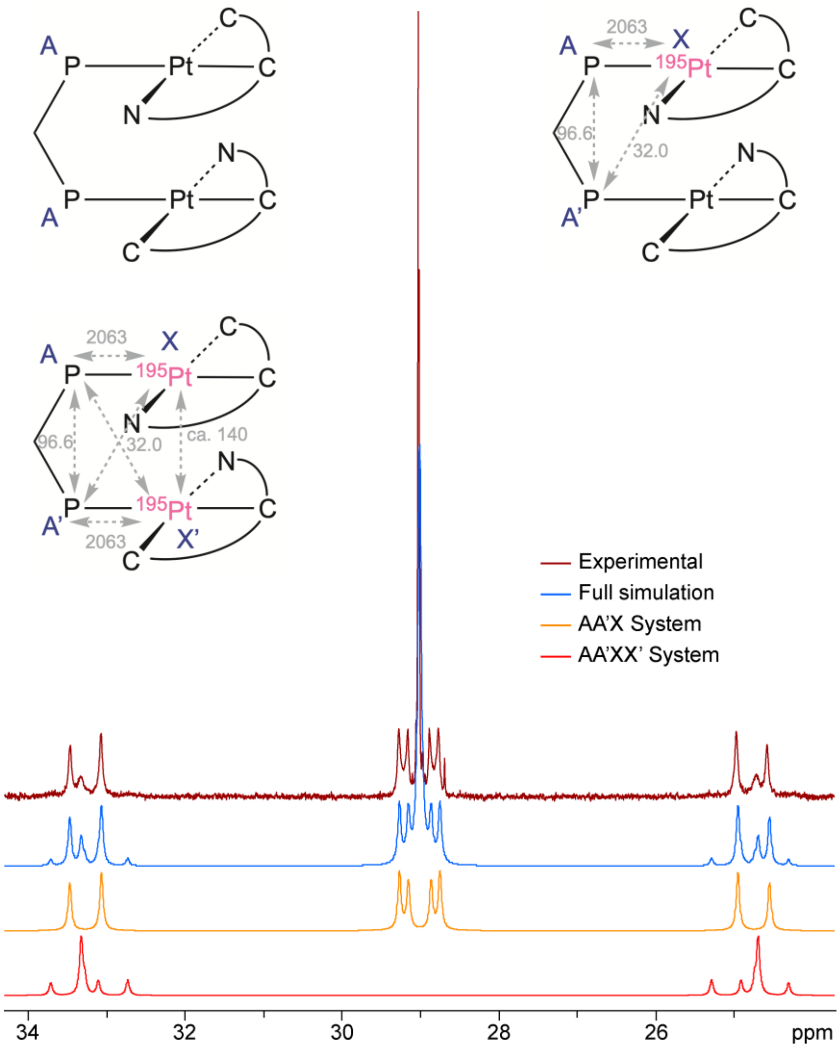

Figure S21. Experimental and simulated $^{31}P\{^{1}H\}$ NMR spectra of **2** (CD_2Cl_2, 243 MHz). Intensities are arbitrary. The three possible isotopologues are represented with the calculated values of the coupling constants in Hz.

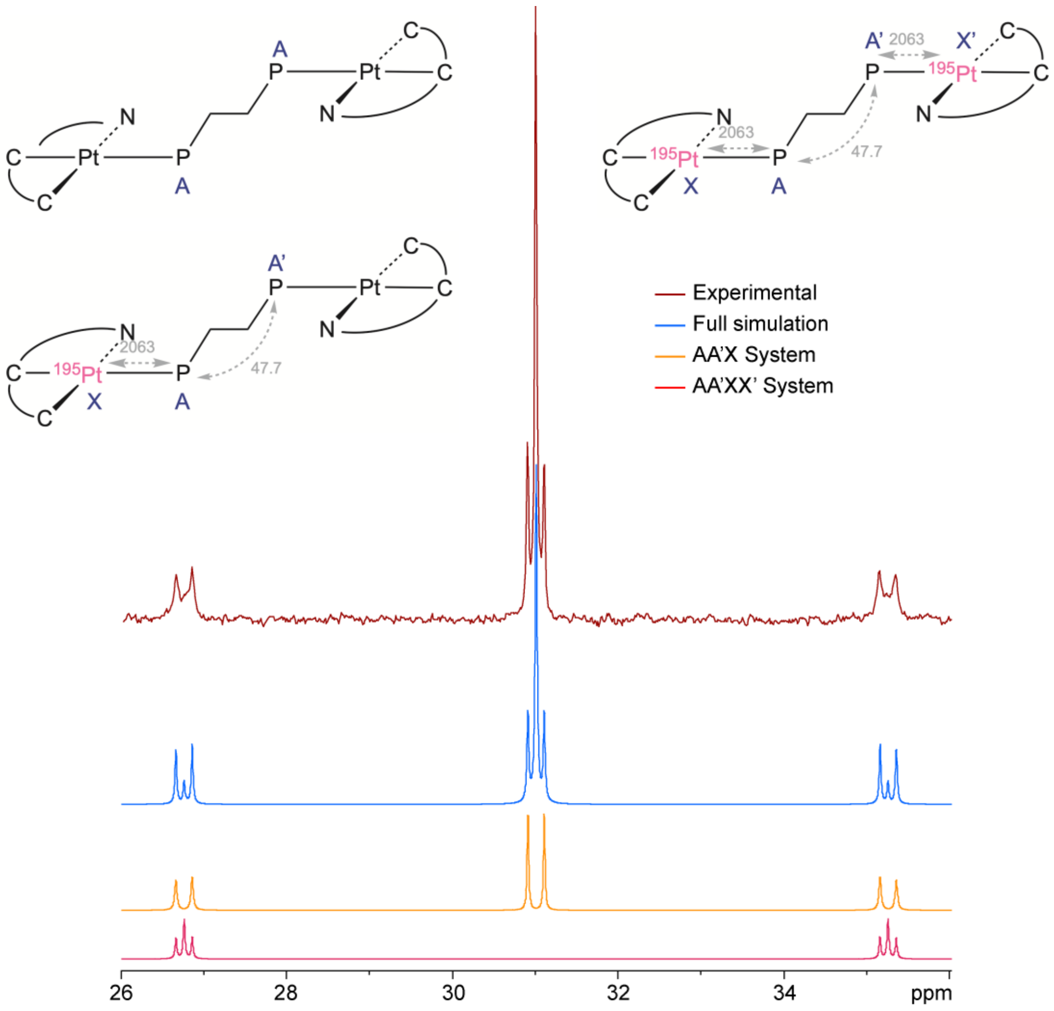

Figure S22. Experimental and simulated $^{31}P\{^{1}H\}$ NMR spectra of **3** (CD_2Cl_2, 243 MHz). Intensities are arbitrary. The three possible isotopologues are represented with the calculated values of the coupling constants in Hz.

4. X-ray structure determinations

Single crystals suitable for X-ray diffraction were grown by slow liquid-liquid diffusion from CH_2Cl_2/*n*-hexane (**2**) or CH_2Cl_2/MeOH (**3**). Diffraction data were collected on a Bruker D8 QUEST diffractometer with monochromated Mo-*K*α radiation performing φ and ω scans. The structures were solved by dual methods and refined anisotropically on F^2 using the program SHELXL-2019 (G. M. Sheldrick, University of Göttingen).[3,4] Numerical details are presented in Table S1. Methyl hydrogens were included as part of rigid idealized methyl groups allowed to rotate but not tip; other hydrogens were included using a riding model. *Special features of refinement:* In **2**, one of the non-metalated *p*-tolyl rings is disordered over two positions, ca 85:15%.

Table S1. Crystallographic data for **2** and **3**.

	**2**	**3**
formula	$C_{75}H_{60}N_2P_2Pt_2$	$C_{76}H_{62}N_2P_2Pt_2$
fw	479.78	1445.39
T (K)	100(2)	100(2)
λ	0.71073	0.71073
cryst syst	Triclinic	Triclinic
space group	P-1	P-1
a (Å)	13.7346(13)	11.0948(4)
b (Å)	14.4447(14)	12.1072(5)
c (Å)	16.1932(15)	13.0718(5)
α (°)	75.443(3)	66.6800(10)
β (°)	67.874(3)	65.657(2)
γ (°)	81.590(3)	88.2410(10)
V (Å^3)	2875.7(5)	1450.56(10)
Z	6	1
ρ_{calcd} (Mg m^{-3})	1.662	1.666
μ (mm^{-1})	4.963	4.920
R1^a	0.0218	0.0147
wR2^b	0.0499	0.0346

^{a}R1 = $\Sigma||F_o| - |F_c||/\Sigma|F_o|$ for reflections with $I > 2\sigma(I)$. ^{b}wR2 = $[\Sigma[w(F_o^2 - F_c^2)^2/\Sigma[w(F_o^2)^2]]^{0.5}$ for all reflections; $w^{-1} = \sigma^2(F^2) + (aP)^2 + bP$, where $P = (2F_c^2 + F_o^2)/3$ and a and b are constants set by the program.

Table S2. Bond distances (Å) and angles (º) selected for **2**.

Pt1-C7	1.979(3)	Pt1-C13	2.051(3)
Pt2-C7A	1.974(3)	Pt2-C13A	2.046(3)
Pt1-N1	2.169(2)	Pt2-N2	2.169(3)
Pt1-P1	2.3354(7)	Pt2-P2	2.3216(8)
P1-C67	1.856(3)	P2-C67	1.844(3)
N1-Pt1-C7	77.45(11)	C7-Pt1-C13	79.50(12)
N1-Pt1-C13	156.95(10)	C7-Pt1-P1	174.07(9)
N1-Pt1-P1	107.46(7)	C67-P1-Pt1	114.00(10)
P1-C67-P2	120.11(16)	C7A-Pt2-C13A	79.75(12)
N2-Pt2-C7A	78.04(11)	C7A-Pt2-P2	174.85(9)
N2-Pt2-C13A	157.53(11)	N2-Pt2-P2	99.87(7)
C67-P2-Pt2	112.49(10)		

Table S3. Bond distances (Å) and angles (º) selected for **3.**

Pt-C1	1.9729(18)	Pt-P1	2.3203(5)
Pt-N1	2.1514(16)	P1-C38	1.8408(19)
Pt-C12	2.0369(18)		
C1-Pt-N1	78.01(7)	C1-Pt-C12	79.98(8)
C12-Pt-N1	157.66(7)	C1-Pt-P1	167.25(6)
C12-Pt-P1	95.90(5)	N1-Pt-P1	106.42(4)
C38-P1-Pt	107.71(6)		

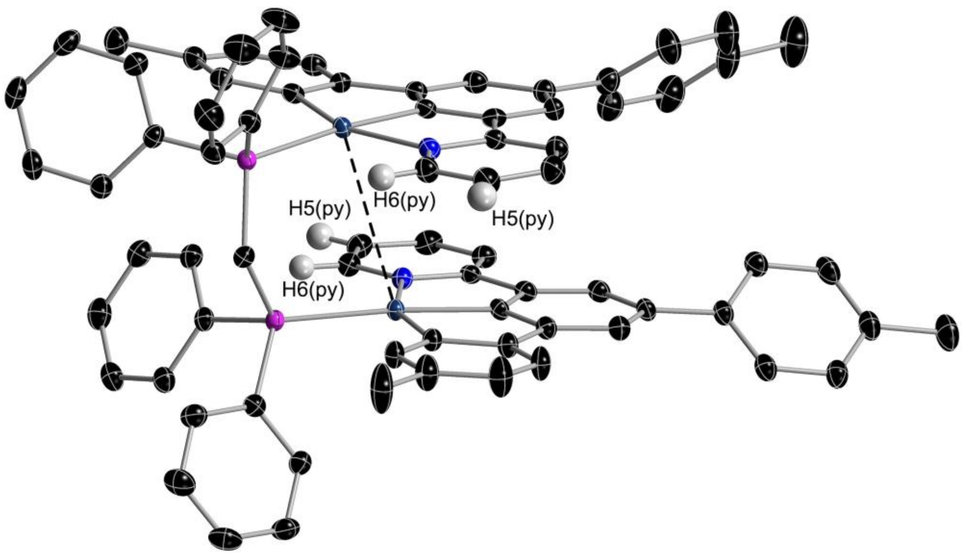

Figure S23. View of the molecule of **2** in the crystal highlighting the H5 and H6 atoms of the pyridyl ring that appear shielded in the ^{1}H NMR spectra (thermal ellipsoids at 50% probability).

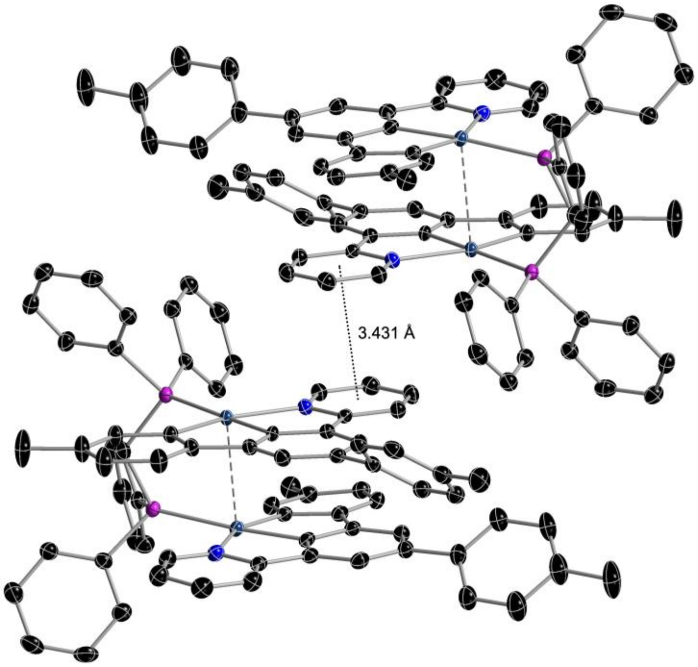

Figure S24. Stacked dimer in the structure of **2** (thermal ellipsoids at 50% probability). Hydrogen atoms and solvent molecules are omitted.

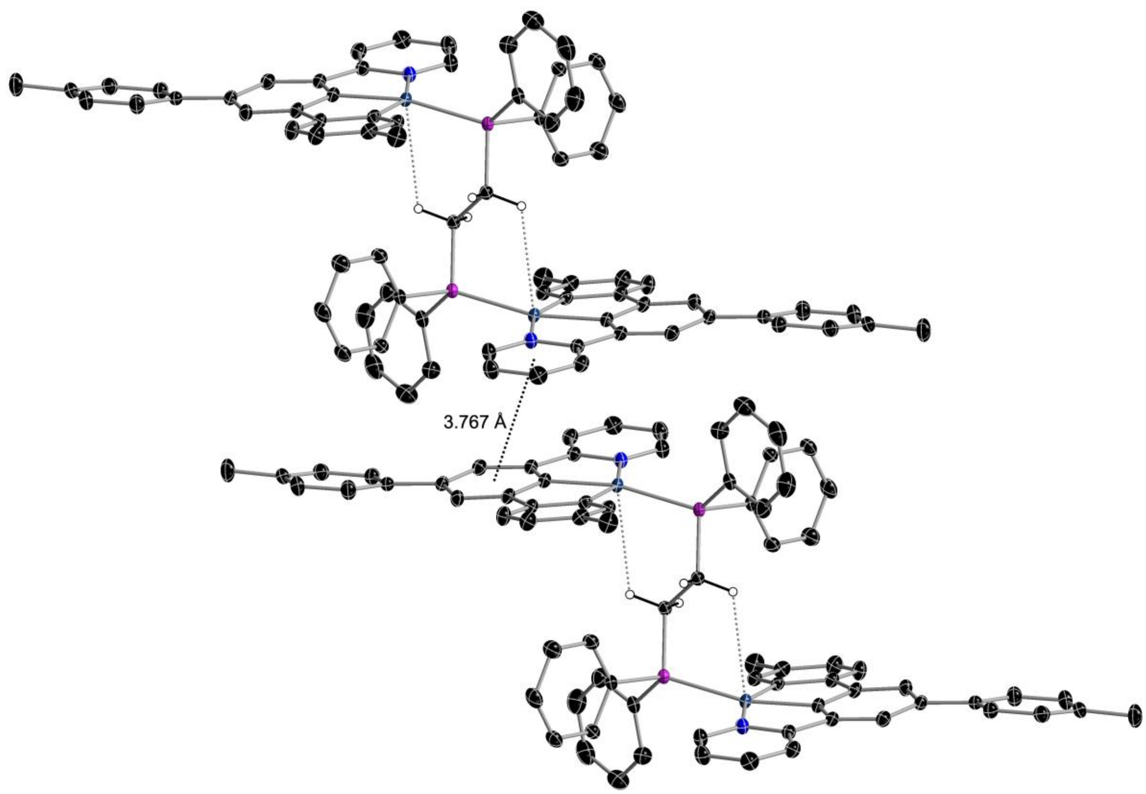

Figure S25. Stacked dimer in the structure of **3** (thermal ellipsoids at 50% probability). Hydrogen atoms and solvent molecules are omitted.

5. Additional photophysical data

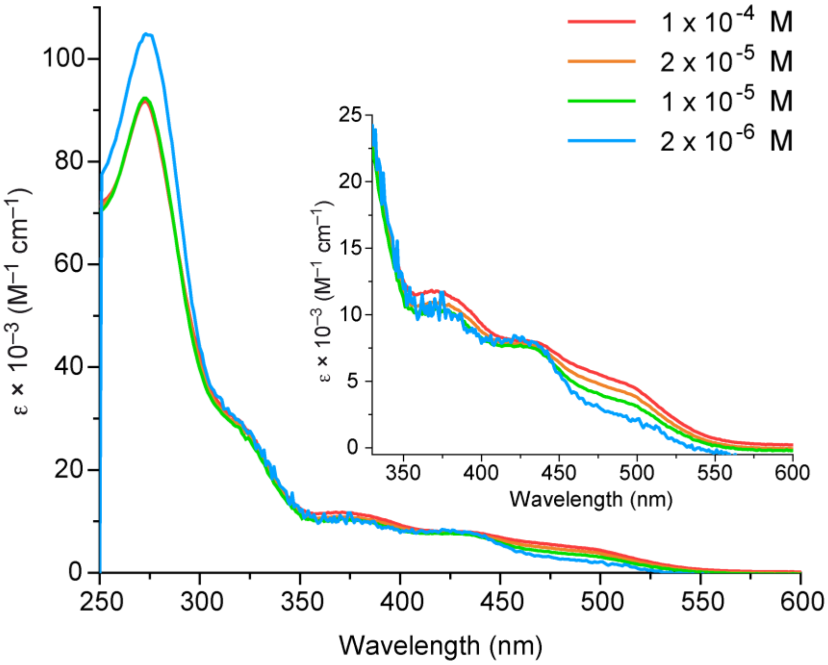

Figure S26. Absorption spectra of **2** in a MeCN solution at different concentrations.

Table S4. Electronic absorption data of complex **2** in different solvents (ca. 1.0×10^{-5} M) at 298 K.

**Solvent**	**λ_{max} /nm ($\varepsilon \times 10^{-3}$ M^{-1} cm^{-1})**
Acetone	376 (11), 437 (8), 500 (4)
MeCN	378 (10), 431 (8), 501 (3)
DMF	377 (11), 437 (7), 498 (4)
Toluene	378 (12), 421 (8), 550 (0.4)
MeOH	380 (11), 442 (8), 492 (4)
Cyclohexane	360 (14), 437 (7), 502 (3)
DMSO	375 (11), 421 (10), 504 (2)

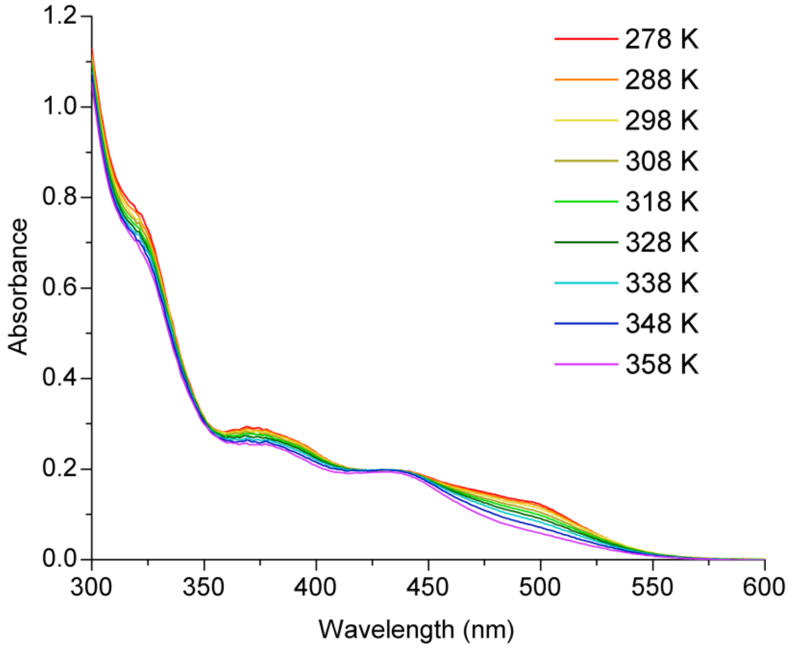

Figure S27. Absorption spectra of **2** in a DMF solution (ca. 2.5×10^{-5} M) at different temperatures.

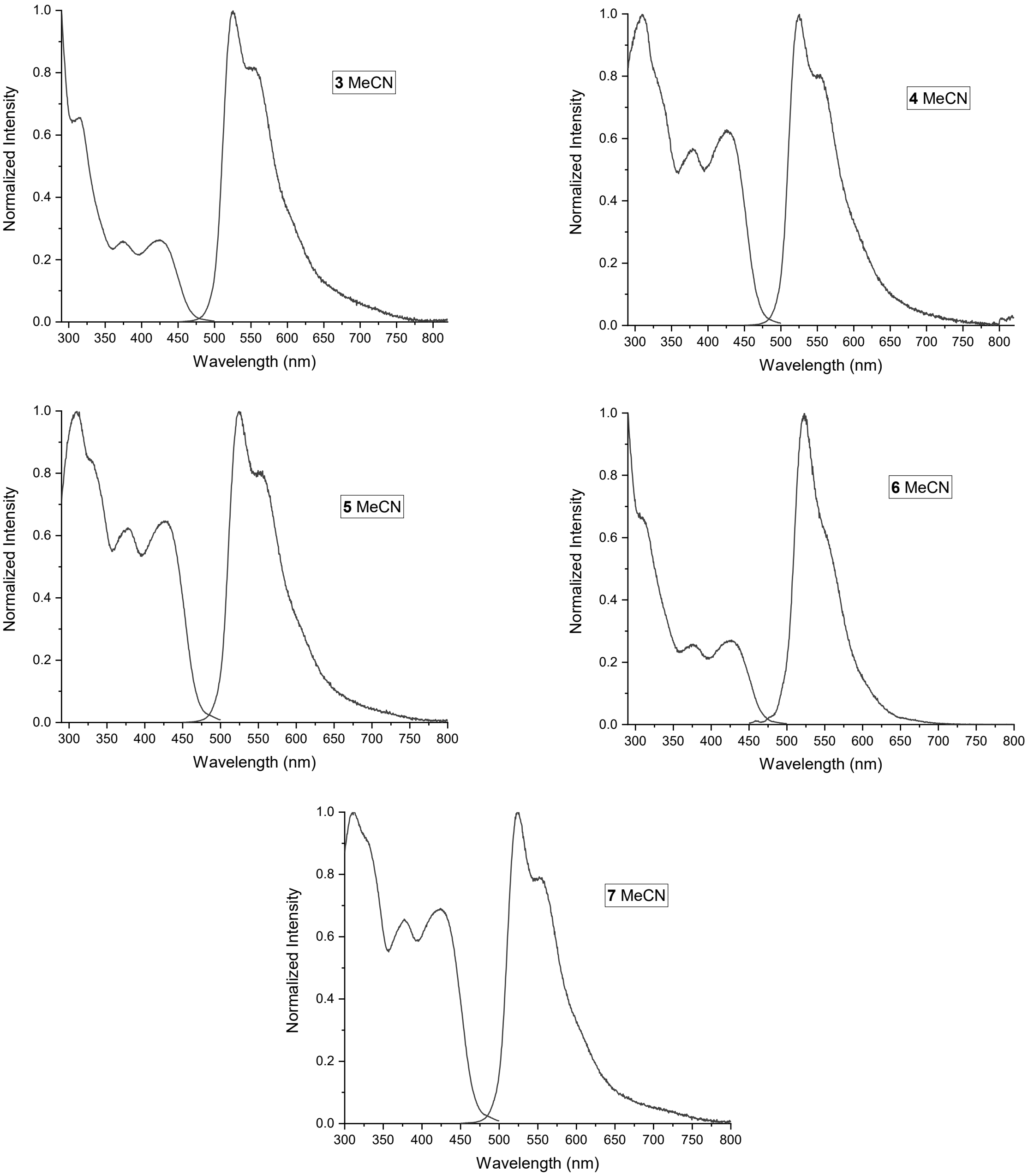

Figure S28. Excitation and emission spectra of complexes **3–7** in MeCN at 298 K. The collected λ_{em} for excitation spectra corresponds in all cases to the highest-energy emission peak.

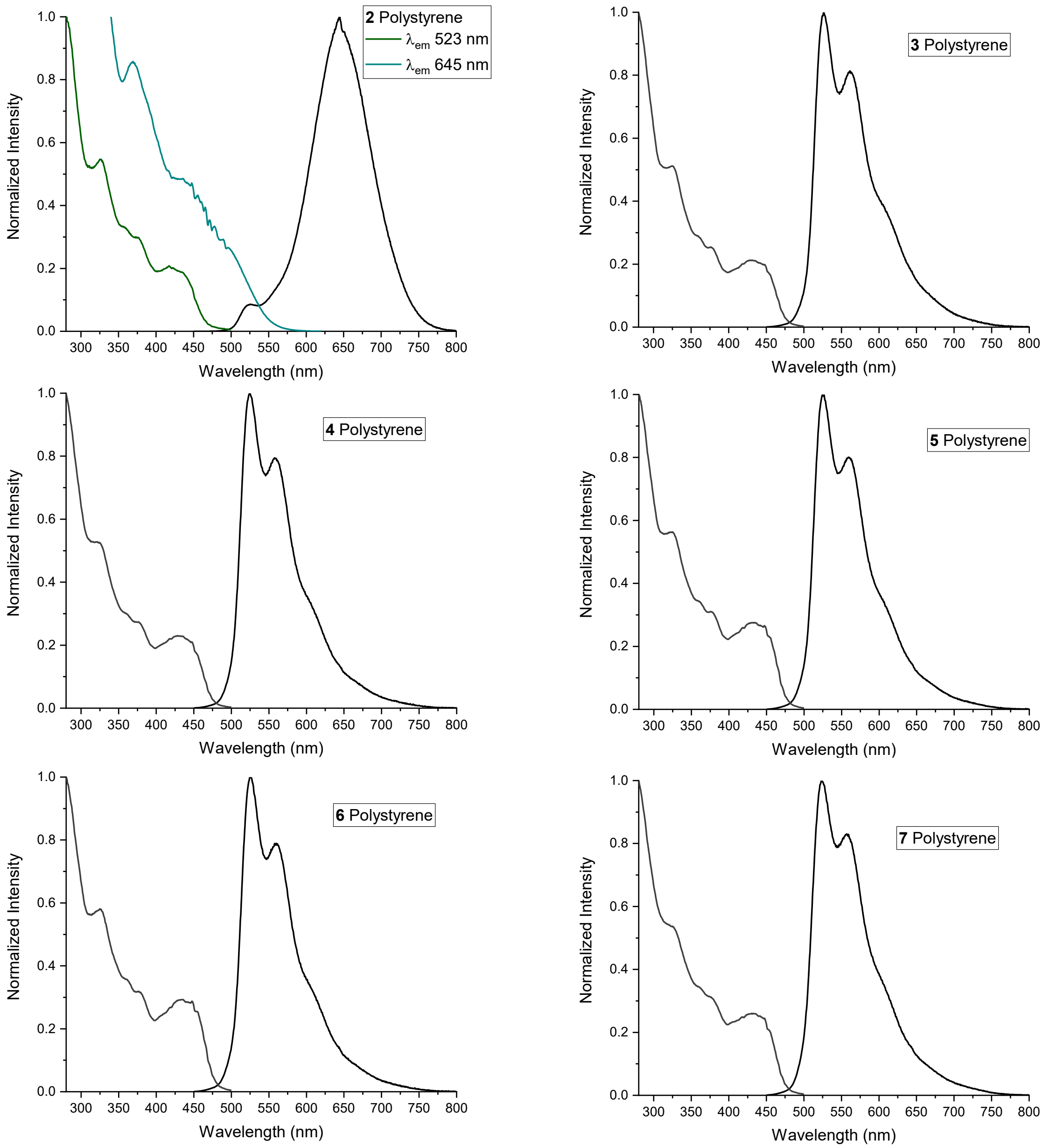

Figure S29. Excitation and emission spectra of complexes **2–7** in polystyrene matrices (2 wt%) at 298 K. The collected λ_{em} for excitation spectra corresponds in all cases to the highest-energy emission peak, with the exception of **2**, for which two emission wavelengths have been monitored.

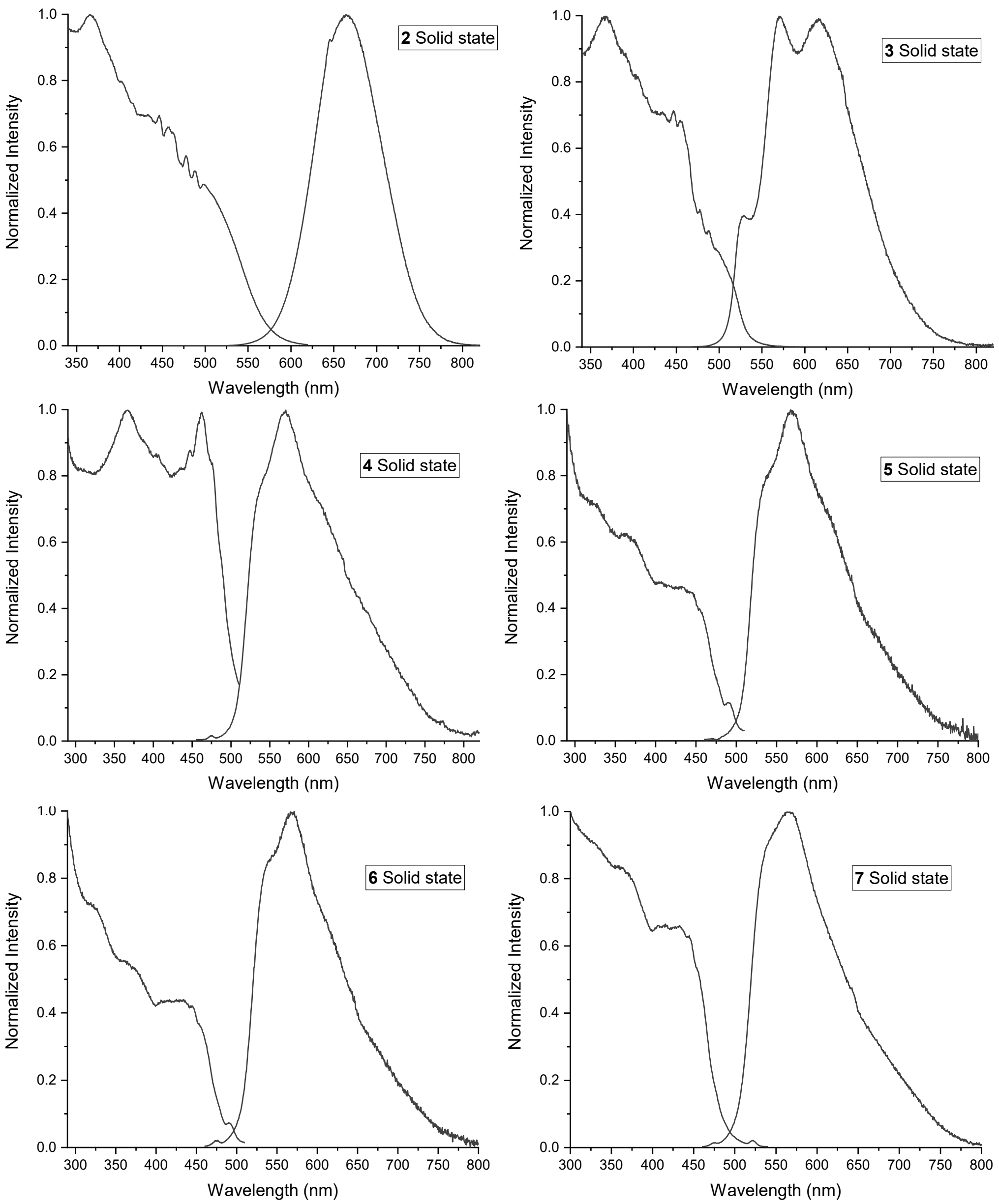

Figure S30. Excitation and emission spectra of complexes **2–7** in the solid state at 298 K. The collected λ_{em} for excitation spectra corresponds in all cases to the highest-energy emission peak.

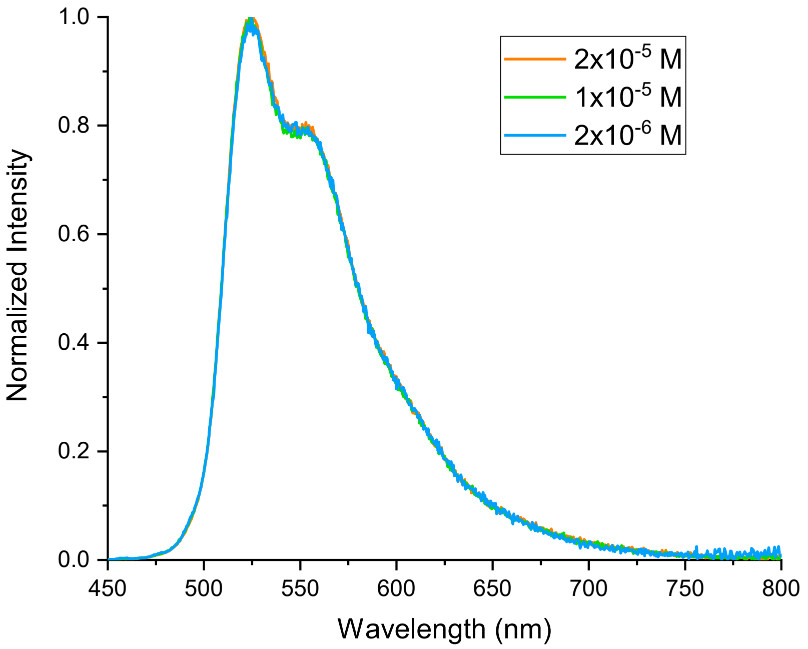

Figure S31. Emission spectra of complex **5** in MeCN at different concentrations.

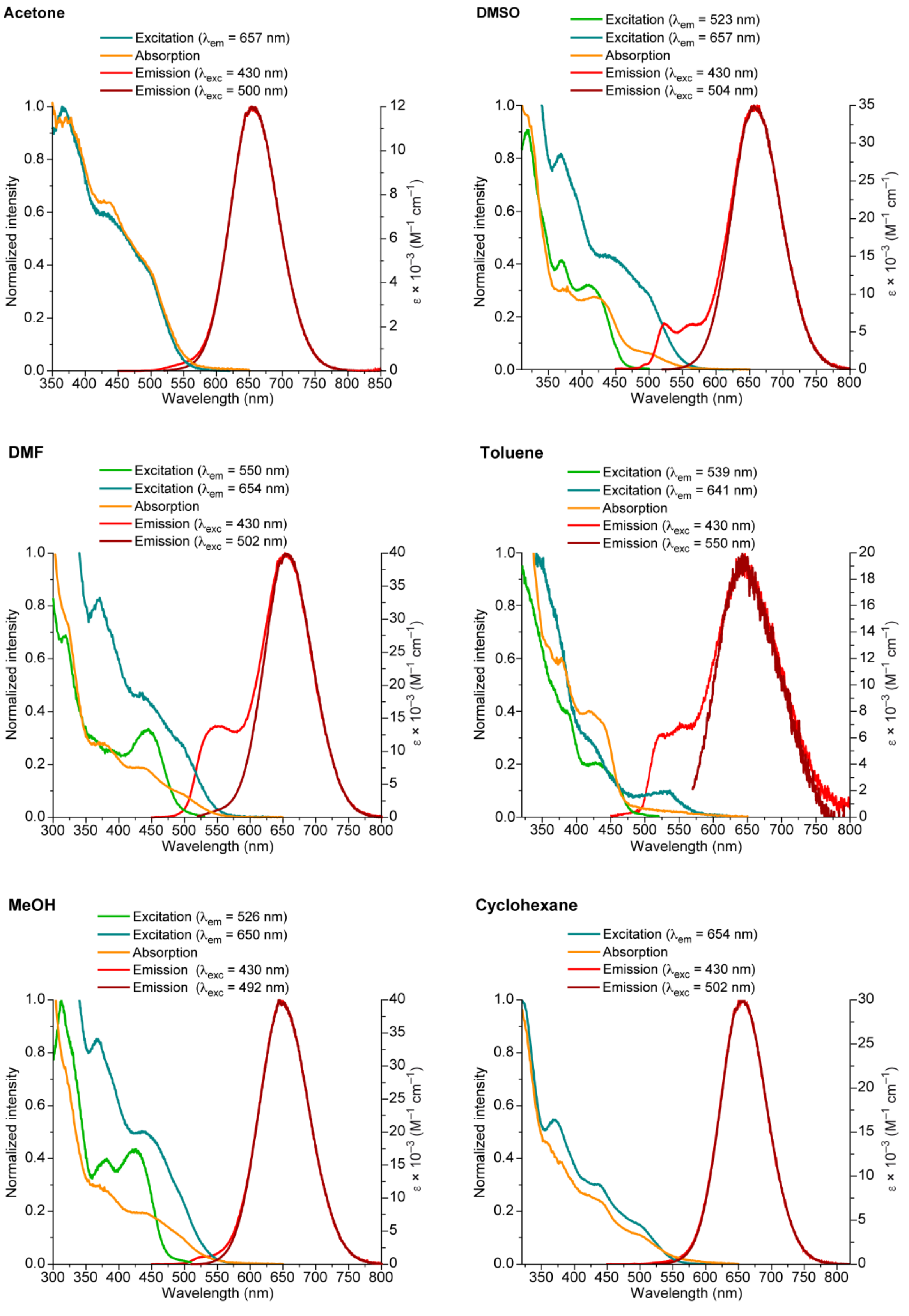

Figure S32. Excitation and emission spectra of complex **2** in different solvents (1.0×10^{-5} M) at 298 K. The absorption spectrum has been included for comparison.

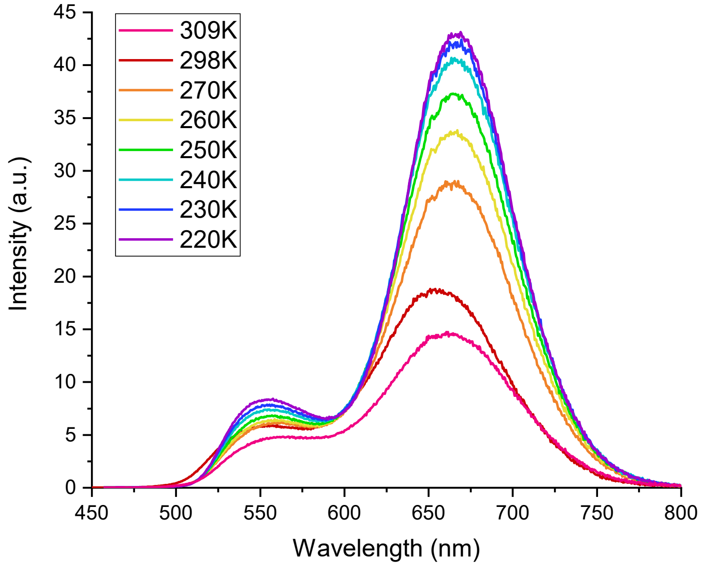

Figure S33. Emission spectra of complex **2** in DMF at different temperatures.

Table S5. Percentage contributions of the emission intensities associated with the open and closed conformations of complex **2** at different temperatures, determined by deconvolution of the emission spectra using the Edgeworth–Cramer peak function (ECS) implemented in OriginPro.

**Temperature (K)**	**Open conformation (% emission)**	**Closed conformation (% emission)**
309	21	79
298	19	81
270	16	84
260	15	85
250	14	86
240	14	86
230	15	85
220	15	85

6. References

(1) Brown, M. P.; Fisher, J. R.; Franklin, S. J.; Puddephatt, R. J.; Seddonf, K. R. 31P NMR Spectra and the Metal—Metal Bond Strength in Some Bis-μ-[Bis(Diphenylphosphino)Methane]Diplatinum Complexes. *J. Organomet. Chem.* **1978**, *161*, 46-C48.

(2) Wrackmeyer, B.; Klimkina, E. V.; Schmalz, T.; Milius, W. Synthesis, NMR Spectroscopic Characterization and Structure of a Divinyldisilazane-(Triphenylphosphine)Platinum(0) Complex: Observation of Isotope-Induced Chemical Shifts $^{1}\Delta^{12/13}C(^{195}Pt)$. *Magn. Reson. Chem.* **2013**, *51*, 283–291.

(3) Sheldrick, G. M. A Short History of SHELX. *Acta Crystallogr., Sect. A: Found. Crystallogr.* **2008**, *64*, 112–122.

(4) Sheldrick, G. M. SHELXT – Integrated Space-Group and Crystal-Structure Determination. *Acta Crystallogr. A Found. Adv.* **2015**, *71*, 3–8.
